# Identification of an arthropod molecular target for plant-derived natural repellents

**DOI:** 10.1073/pnas.2118152119

**Published:** 2022-04-22

**Authors:** Quan Tian, Peiyu Wang, Chang Xie, Peiyuan Pang, Youjing Zhang, Yue Gao, Zhijian Cao, Yingliang Wu, Wenxin Li, Michael X. Zhu, Dongdong Li, Jing Yao

**Affiliations:** ^a^State Key Laboratory of Virology, College of Life Sciences, Wuhan University, Wuhan, Hubei 430072, China;; ^b^Hubei Key Laboratory of Cell Homeostasis, College of Life Sciences, Wuhan University, Wuhan, Hubei 430072, China;; ^c^Department of Anesthesiology, Zhongnan Hospital of Wuhan University, Wuhan, Hubei 430072, China;; ^d^Frontier Science Center for Immunology and Metabolism, Wuhan University, Wuhan, Hubei 430072, China;; ^e^Modern Virology Research Center, College of Life Sciences, Wuhan University, Wuhan, Hubei 430072, China;; ^f^Department of Integrative Biology and Pharmacology, McGovern Medical School, The University of Texas Health Science Center at Houston, Houston, TX 77030;; ^g^Sorbonne Université, Institute of Biology Paris Seine, Neuroscience Paris Seine, CNRS UMR8246, INSERM U1130, Paris 75005, France

**Keywords:** scorpion, TRP channels, repellents, avoidance behavior

## Abstract

Arthropods maintain ecosystem balance while also contributing to the spread of disease. Plant-derived natural repellents represent an ecological method of pest control, but their direct molecular targets in arthropods remain to be further elucidated. Occupying a critical phylogenetic niche in arthropod evolution, scorpions retain an ancestral genetic profile. Here, using a behavior-guided screening of the *Mesobuthus martensii* genome, we identified a scorpion transient receptor potential (sTRP1) channel that senses *Cymbopogon*-derived natural repellents, while remaining insensitive to the synthetic chemical pesticide DEET. Scrutinizing orthologs of sTRP1 in *Drosophila melanogaster*, we further demonstrated dTRPγ ion channel as a chemosensory receptor of natural repellents to mediate avoidance behavior. This study sheds light on arthropod molecular targets of natural repellents, exemplifying the arthropod–plant adaptation. It should also help the rational design of insect control strategy and in conserving biodiversity.

## Significance

Rational control of arthropod pests is important for animal and human health as well as biodiversity preservation. As an alternative to synthetic chemical pesticides, natural repellents represent an ecological method of pest control. Through an exceptional gene library screening in *Mesobuthus martensii* scorpions, we here uncover a transient receptor potential ion channel as the chemosensory sensor for plant-derived repellents. Its ortholog ion channel in *Drosophila melanogaster* also acts as a molecular receptor of natural repellents and mediates avoidance behavior. This work thus identifies a molecular basis for arthropod chemosensing and should help update the ecological strategies for pest control while preserving biodiversity.

Arthropods are invertebrates that arose during the Cambrian period, representing an important component of the ecosystem (1–3). Albeit indispensable for maintaining the animal food chain, they also spread diseases between animals and humans (4, 5). Synthetic chemical pesticides have been widely used for pest control but cause the decline of arthropod populations (6) and also pose health concerns to humans (7–9). Natural repellents derived from plants stand as an alternative and ecological strategy for pest control (10, 11). The avoidance to natural repellents also reflects an evolutionary adaptation between arthropods and plants, which contributes to the maintenance of ecosystem homeostasis. The molecular basis underlying the reaction of arthropods to natural repellents, however, remains to be further understood. Conserving the primary genomic characters of Paleozoic ancestors from the Cambrian Age, scorpions are witnesses to arthropod evolution (12). Deep transcriptome analyses reveal that the *Mesobuthus martensii* genome comprises the most protein-encoding genes among all sequenced arthropods (13). *M. martensii*, therefore, represents an exploitable model for searching for molecular targets of specific repellents.

Here, we performed a mesoscale screening of the *M. martensii* genome with behavioral and functional assays. Our data unveil a transient receptor potential (TRP) ion channel, sTRP1, that specifically responds to the natural repellents citronellal and citronellol extracted from *Cymbopogon citratus* and mediates the animal’s avoidance behavior. In contrast, sTRP1 is insensitive to the synthetic chemical pesticide DEET. Interestingly, camphor, an extract of camphor laurel (*Cinnamomum camphora*), is also a ligand of sTRP1. Further functional and genetic characterization in *Drosophila melanogaster* demonstrates that dTRPγ, the sTRP1 ortholog in flies, also acts as a molecular receptor of plant-derived repellents. Our data provide molecular insights into the evolutionary adaptation between arthropods and plants and help to design ecological strategies for pest control and biodiversity conservation.

## Results

### Natural Repellents Cause *M. martensii* Avoidance Behavior via Depolarizing Ventral Nervous Cord Ganglion Neurons.

**Fig. 1.**
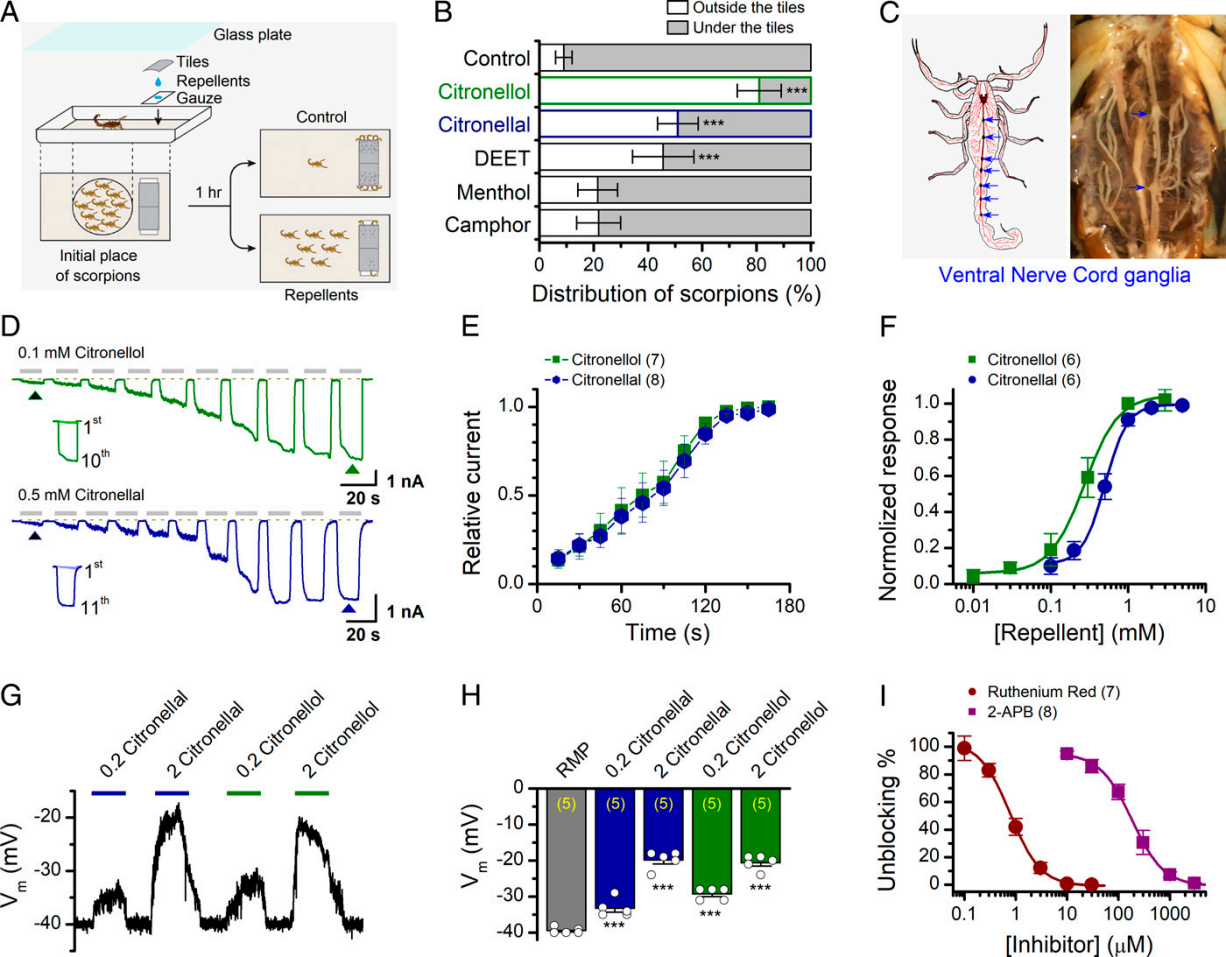
Repellents citronellal and citronellol stimulate scorpion avoidance response via depolarizing its VNC ganglion neurons. (*A*) Schematic representation of scorpion avoidance response assay. Two pieces of gauze were preimmersed with water (control) or a solution with the desired concentration of the test drug and placed at one side of the container (100 cm × 50 cm × 30 cm) with a layer of clean sand. Each gauze was covered by a piece of clay tile. For each trial, 100 scorpions were placed at the center of the container covered with a piece of glass. After about 1 h, the numbers of scorpions under and outside the tiles were separately counted. (*B*) Summary of the assay shown in *A*. Note, in the citronellal and citronellol groups, >50% of scorpions were outside of the tiles. Data from five or six independent experiments per group are presented. Error bars indicate SD. ***P = 2.56E−5 for citronellal, 2.70E−8 for citronellol, and 5.23 E−6 for DEET; *P* = 0.106 for camphor and 0.120 for menthol versus control (ANOVA). (*C*) Schematic diagram of scorpion VNC showing a total of seven ganglia (*Left*) and an example anatomical picture (*Right*). (*D*) Representative whole-cell recordings of scorpion VNC neurons in response to repeated applications of citronellol (0.1 mM, *Top*) and citronellal (0.5 mM, *Bottom*), respectively. Each drug application lasted for ∼15 s. Holding potential (V_h_) was −60 mV. A comparison between the responses of cells to the first and the 10th applications is shown in *Inset*. The dotted line indicates zero current level. (*E*) Time courses of peak currents elicited by repeated applications of citronellol (*n* = 7) and citronellal (*n* = 8). Currents were normalized by the maximum values after sensitization. (*F*) Concentration-response curves of citronellal and citronellol after full sensitization. The solid lines are fits to the Hill equation with EC_50_ = 0.27 ± 0.02 mM, n_H_ = 2.0 ± 0.2 for citronellol (*n* = 6); and EC_50_ = 0.50 ± 0.02 mM, n_H_ = 3.0 ± 0.4 for citronellal (*n* = 6). (*G*) Representative current-clamp responses of isolated scorpion VNC neurons consecutively challenged with citronellal (0.2 and 2 mM) and citronellol (0.2 and 2 mM). Note, the injected current was set to zero. (*H*) Statistics plot of membrane potential. The addition of different concentrations of citronellal or citronellol caused neurons to undergo different degrees of depolarization. RMP, resting membrane potential. Number of cells is indicated in parentheses. ***P = 3.38E−4 for 0.2 mM citronellal, 2.57E−8 for 2 mM citronellal, 4.53E−7 for 0.2 mM citronellol, 2.57E−8 for 2 mM citronellol versus RMP (ANOVA). (*I*) Concentration-response curves of inhibitory effects of RR and 2-APB on isolated VNC neurons in the presence of citronellol (2 mM). Solid lines indicate fits with the Hill equation, which yielded IC_50_ = 0.78 ± 0.02 μM, n_H_ = 1.5 ± 0.1 for RR (*n* = 7); and IC_50_ = 182.45 ± 13.15 μM, n_H_ = 1.4 ± 0.1 for 2-APB (*n* = 8). Error bars represent SEM.

Harboring abundant genomic footprints of arthropods, scorpions display distinct sensitivity to environmental cues. To test the chemosensory response of *M. martensii* to repellents, we designed an avoidance response assay making use of the behavior preference of scorpions to hide under the shade—in this case, a clay tile from an old village house (Fig. 1*A* and Movie S1). In the absence of any repellent (control), most scorpions stayed under the tile 1 h after being placed in the apparatus (Fig. 1*A* and Movie S1). Distinct repellents were then applied to a gauze placed under the tile to evaluate their effect on scorpion behavior. The natural repellents citronellal and citronellol, extracted from *Cymbopogon* (10), effectively prevented scorpions from entering the tiles (Fig. 1*B*). Notably, the synthetic pesticide DEET also showed a comparable effect to citronellal.

We then assessed the neuronal regulation of the natural repellent–induced avoidance response. As environmental sensing involves a peripheral sensory pathway, we isolated ventral nervous cord (VNC) neurons from *M. martensii* (14) and performed whole-cell patch clamp recording (Fig. 1*C*). Both citronellal and citronellol evoked inward currents in individual VNC neurons held at −60 mV. Notably, the current amplitude gradually increased upon repetitive drug application, reaching a plateau of ∼11-fold of the initial response (Fig. 1 *D* and *E*). The concentration response of VNC neurons was determined after the current had stabilized, which yielded EC_50_ values of 0.50 ± 0.02 mM (n_H_ = 3.0 ± 0.4, *n* = 6) for citronellal and 0.27 ± 0.02 mM (n_H_ = 2.0 ± 0.2, *n* = 6) for citronellol (Fig. 1*F*). In current-clamp mode, applications of citronellal or citronellol resulted in membrane depolarization of VNC neurons (Fig. 1 *G* and *H*), thereby facilitating their excitability. The current and voltage response patterns (Fig. 1 *D* and *G*) of VNC neurons resemble the use-dependent sensitization of certain mammalian TRP channels (15–17). Indeed, we found that the whole-cell currents evoked by citronellal were inhibited by TRP channel inhibitors ruthenium red (RR) and 2-aminoethoxydiphenyl borate (2-APB) in a concentration-dependent manner (Fig. 1*I*). For 2 mM citronellal, the IC_50_ was 0.78 ± 0.02 μM (n_H_ = 1.5 ± 0.1, *n* = 7) for RR and 182.45 ± 13.15 μM (n_H_ = 1.4 ± 0.1, *n* = 8) for 2-APB. These results suggest that a natural repellent–caused avoidance response in *M. martensii* is likely mediated by a TRP channel.

### Identification of a Natural Repellent–Sensing Receptor in *M. martensii*.

TRP ion channels act as multimodal receptors, scrutinizing environmental cues to guide animal behavior (18). We then screened putative *trp* genes from the complementary DNA (cDNA) library of *M. martensii* using the RACE (rapid amplification of cDNA ends) PCR technique (15, 17, 19) with primers designed according to the conserved motifs of mammalian TRP channel sequences. We identified and cloned six putative genes and named them scorpion TRP1–6 (*strp1–6*). To determine if these genes encode functional channels that are responsive to citronellal and citronellol, we transiently expressed each cDNA in human embryonic kidney (HEK) 293T cells and performed whole-cell patch clamp recording. We found that only cells expressing the *sTRP1* cDNA responded to citronellal and citronellol (*SI Appendix*, Fig. S1).

**Fig. 2.**
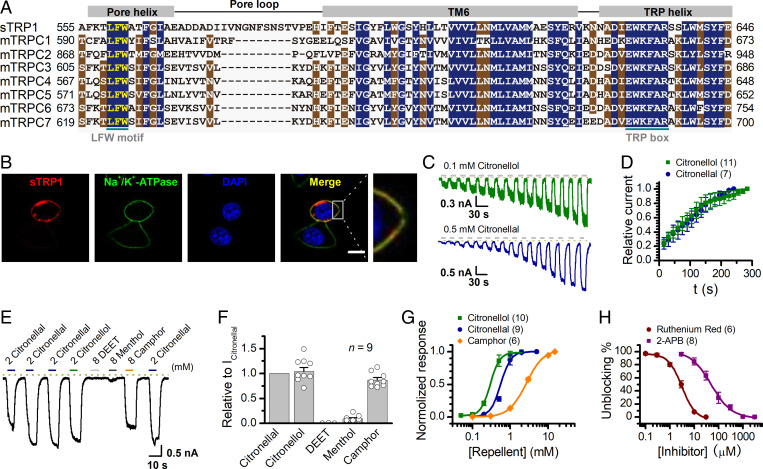
sTRP1 channel acts as a natural repellent receptor. (*A*) Amino acid sequence alignments of sTRP1 and mouse TRPC1-7 within pore region, TM domain 6 (TM6), and TRP helix. Residues that are identical or similar among the sequences are shaded in blue and brown, respectively. (*B*) Distribution of sTRP1-mCherry protein (red) in transfected HEK 293T cells assessed by confocal microscopy. Na^+^/K^+^-ATPase (green) was labeled by antibodies to indicate where the cell membrane is, and nucleus (blue) was stained by DAPI. (*C*) Representative whole-cell recordings from sTRP1-expressing HEK 293T cells evoked by repeated applications of citronellol (0.1 mM) or citronellal (0.5 mM). V_h_ = −60 mV. (*D*) Statistical plot of relative current of peak response over repetitive stimulation. Data were normalized to the last pulse response (*n* = 11 for citronellol, *n* = 7 for citronellal). (*E*) Representative whole-cell recordings of sTRP1-expressing HEK 293T cells that responded to variable repellents after full sensitization. (*F*) Summary plot of relative response. Currents were normalized to the current elicited by 2 mM citronellal (*n* = 9). (*G*) Concentration-response curves of citronellal, citronellol, and camphor activation of sTRP1 channels. Solid lines indicate fits to the Hill equation with EC_50_ = 0.56 ± 0.01 mM, n_H_ = 3.3 ± 0.5 for citronellal (*n* = 9); EC_50_ = 0.30 ± 0.01 mM, n_H_ = 3.6 ± 0.3 for citronellol (*n* = 10), and 3.3 ± 0.2 mM, n_H_ = 2.4 ± 0.4 for camphor (*n* = 6). (*H*) Dose-response curves of inhibitory effects of RR and 2-APB on sTRP1 in the presence of 2 mM citronellal after sensitization. The solid lines correspond to fits by the Hill equation with IC_50_ = 3.12 ± 0.29 μM, n_H_ = 1.6 ± 0.2 for RR (*n* = 6); and IC_50_ = 48.32 ± 5.58 μM, n_H_ = 1.3 ± 0.1 for 2-APB (*n* = 8). The dotted line indicates zero current level. Error bars represent SEM.

Scorpion *trp1* encodes a protein consisting of 791 amino acids (*SI Appendix*, Fig. S2). The presence of an EWKFAR motif at its putative C terminus (Ct) and the analysis of the phylogenetic tree suggest that sTRP1 belongs to the canonical TRP (TRPC) family (*SI Appendix*, Fig. S3). An alignment of the predicted sequence encompassing the putative pore helix, S6 transmembrane (TM) segment, and the TRP helix revealed that sTRP1 and all seven mammalian TRPC members share ∼61% overall identity in these regions (Fig. 2*A*). By comparing with human TRPC3 and TRPC6 channels, of which single-particle cryo-electron microscopic structures are available (20), other conserved structural features of TRPC channels (e.g., four ankyrin-like repeats and six TM segments) were also readily identified in sTRP1 (*SI Appendix*, Fig. S2). Using the qPCR assay, we confirmed expression of sTRP1 throughout the body of *M. martensii* (*SI Appendix*, Fig. S4).

We further characterized the channel property of sTRP1 heterologously expressed in HEK 293T cells. As illustrated in Fig. 2*B*, sTRP1, along with the membrane marker Na^+^/K^+^-ATPase, was well expressed on the cell surface. As with the isolated scorpion VNC neurons, citronellal evoked inward whole-cell currents at −60 mV that progressively increased upon repetitive stimulation (Fig. 2 *C* and *D*). Additionally, sTRP1 responded to the natural repellents camphor and menthol, although the response was smaller. Interestingly, sTRP1 showed no response to the synthetic chemical pesticide DEET (Fig. 2 *E* and *F*). The concentration-response relationships determined after reaching the full sensitization revealed EC_50_ values of 0.56 ± 0.01 mM (n_H_ = 3.3 ± 0.5, *n* = 9) for citronellal, 0.30 ± 0.01 mM (n_H_ = 3.6 ± 0.3, *n* = 10) for citronellol, and 3.3 ± 0.2 mM (n_H_ = 2.4 ± 0.4, *n* = 6) for camphor (Fig. 2*G*). The weak affinity of camphor and menthol for sTRP1 might underlie their low repelling effect on *M. martensii* (Fig. 1*B*). Furthermore, current evoked by 2 mM citronellal was fully inhibited by TRP channel blockers RR (IC_50_ values of 3.12 ± 0.29 μM, n_H_ = 1.6 ± 0.2, *n* = 6) and 2-APB (48.32 ± 5.58 μM, n_H_ = 1.3 ± 0.1, *n* = 8) (Fig. 2*H*), similar to the observation in scorpion VNC neurons.

**Fig. 3.**
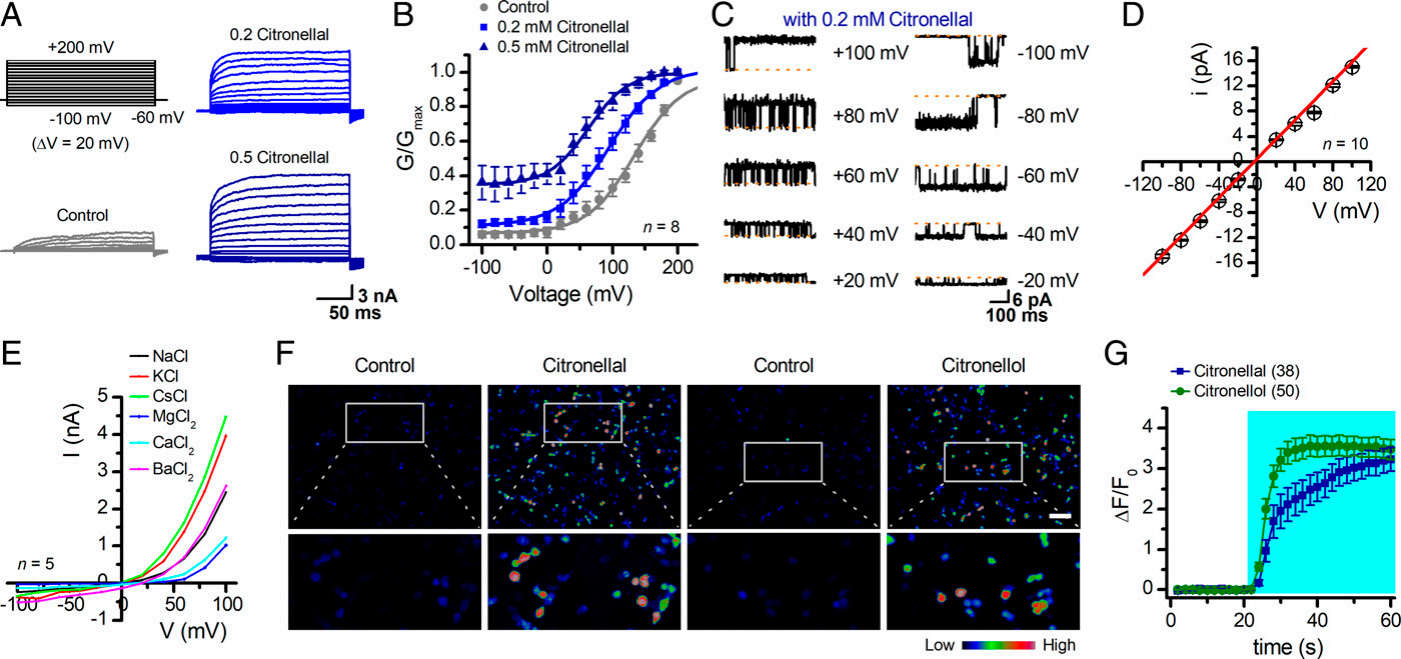
Biophysical properties of sTRP1 channel. (*A*) Representative whole-cell currents of sTRP1-expressing HEK 293T cells elicited by a family of voltage pulses ranging from −100 mV to 200 mV with a 20-mV increment as indicated at upper left, in the presence of normal bath solution (control), 0.2 mM citronellal, or 0.5 mM citronellal. Note, the channels were presensitized by repeated applications of 2 mM citronellal. V_h_ = −60 mV. (*B*) G-V relationships derived from the recordings shown in *A*. Solid lines correspond to fits with Boltzmann function, yielding V_1/2_ = 134.0 ± 3.4 mV and κ = 36.1 ± 3.2, gating charge = 0.71 ± 0.06 for control (*n* = 8); V_1/2_ = 94.6 ± 1.8 mV and κ = 38.6 ± 1.5, gating charge = 0.66 ± 0.02 for 0.2 mM citronellal (*n* = 8), and V_1/2_ = 62.8 ± 2.9 mV and κ = 30.5 ± 2.7, gating charge = 0.84 ± 0.07 for 0.5 mM citronellal (*n* = 5). (*C*) Single-channel currents of sTRP1 recorded from outside-out membrane patches of HEK 293T cells evoked by 0.2 mM citronellal at the indicated holding potentials after sensitization. Sensitization was induced with 2 mM citronellal. Dotted lines indicate the closed channel state. (*D*) Plot of unitary current amplitudes versus voltages. Note that the unitary currents were determined by fitting all-point histograms with Gaussians. Unitary conductance measured by fitting a linear function was 152.9 ± 1.5 pS (*n* = 10). (*E*) Current-voltage relations. Currents were elicited with 100-ms test pulses ranging from −100 mV to +100 mV at an increment of 20 mV for the same cell exposed to different extracellular solutions containing 0.5 mM citronellal and varying cations as indicated (*n* = 5). Pipette solutions contained 140 mM NaCl. (*F*) [Ca^2+^]_i_ increases elicited by different agonists. Responses of sTRP1-expressing HEK 293T cells to 2 mM citronellal or 2 mM citronellol measured by GCaMP6m fluorescence with 1.8 mM extracellular Ca^2+^. Color bar indicates the calibration of intracellular calcium concentration, [Ca^2+^]_i_. Activation of sTRP1 resulted in the rise of [Ca^2+^]_i_. Images showing the levels of [Ca^2+^]_i_ in HEK 293T cells expressing sTRP1 and GCaMP6m both at rest and in response to citronellal and citronellol, respectively. (Scale bar, 100 μm.) (*G*) Time courses of the relative change of fluorescence were plotted from the images shown in *F*. Error bars indicate SEM.

When unstimulated, the sTRP1 channel exhibited weak voltage-dependent activation, showing currents only at positive potentials (V_1/2_ = 134.0 ± 3.4 mV and κ = 36.1 ± 3.2, *n* = 8). In the presence of citronellal, V_1/2_ was left shifted (V_1/2_ = 94.6 ± 1.8 mV and κ = 38.6 ± 1.5, *n* = 8 for 0.2 mM citronellal; V_1/2_ = 62.8 ± 2.9 mV and κ = 30.5 ± 2.7, *n* = 5 for 0.5 mM citronellal) (Fig. 3 *A* and *B*). In addition, the voltage-independent fraction of the conductance was remarkably increased in 0.5 mM citronellal (Fig. 3*B*). In excised patches from sTRP1-expressing HEK 293T cells, we observed unitary currents in the presence of 0.2 mM citronellal, demonstrating its direct activation independently of the cytoplasmic components (Fig. 3*C*). The unitary currents displayed a linear current-voltage (I-V) relationship and a slope conductance of 152.9 ± 1.5 pS (*n* = 10; Fig. 3*D*). Ion substitution experiments performed in whole-cell mode showed that the sTRP1 channel is more selective for divalent than monovalent cations while having no discrimination among Na^+^, K^+^, and Cs^+^ (Fig. 3*E* and *SI Appendix*, Fig. S5). Ca^2+^ imaging using coexpressed GCaMP6m further showed that citronellal and citronellol caused marked increases in intracellular Ca^2+^ levels only in sTRP1-expressing cells (Fig. 3 *F* and *G*), confirming its permeability to Ca^2+^ (Fig. 3 *F* and *G*), a property common to TRPC channels (21). These data show that *M. martensii* sTRP1 acts as a molecular receptor for plant-derived natural repellents.

### *Drosophila* sTRP1 Ortholog, dTRPγ, Is a Natural Repellent Receptor.

The identification of sTRP1 raises the possibility that its homologs in other arthropods might also behave as natural repellent receptors. In another arthropod model, *D. melanogaster*, sTRP1 exhibits the greatest homology to dTRPγ, dTRP-like, and dTRP, which all belong to the TRPC subfamily (*SI Appendix*, Fig. S3). We further compared these TRP subtypes with the full-length amino acid sequence of sTRP1 using the standard protein basic local alignment search tool (BLASTP) program (NCBI). A slightly better E value (2 × 10^−96^, a parameter indicating the “expect” chance of seeing the hit in the database search) was observed for dTRPγ than dTRP-like (1 × 10^−93^) and dTRP (7 × 10^−78^). When comparing the most conserved regions encompassing the pore helix (*SI Appendix*, Fig. S6), the three *Drosophila* TRPs showed comparable similarity to sTRP1 (∼46% identical, ∼64% similar, 10% gaps, out of 100 amino acids).

To determine if any of the *Drosophila* TRPs may be a functional ortholog of sTRP1, we expressed dTRP, dTRP-like, and dTRPγ individually in HEK 293T cells and performed whole-cell recording. As controls, we also expressed other subfamily members of *Drosophila* TRPs in parallel. All 13 *Drosophila* TRP proteins distributed on the cell surface and colocalized with the membrane marker Na^+^/K^+^-ATPase (*SI Appendix*, Fig. S7). However, only the expression of dTRPγ, and not other *Drosophila* TRPs including dTRP and dTRP-like, led to current development in response to the natural repellents citronellal and citronellol (2 mM; *SI Appendix*, Fig. S7), indicating that dTRPγ is a functional ortholog of sTRP1.

**Fig. 4.**
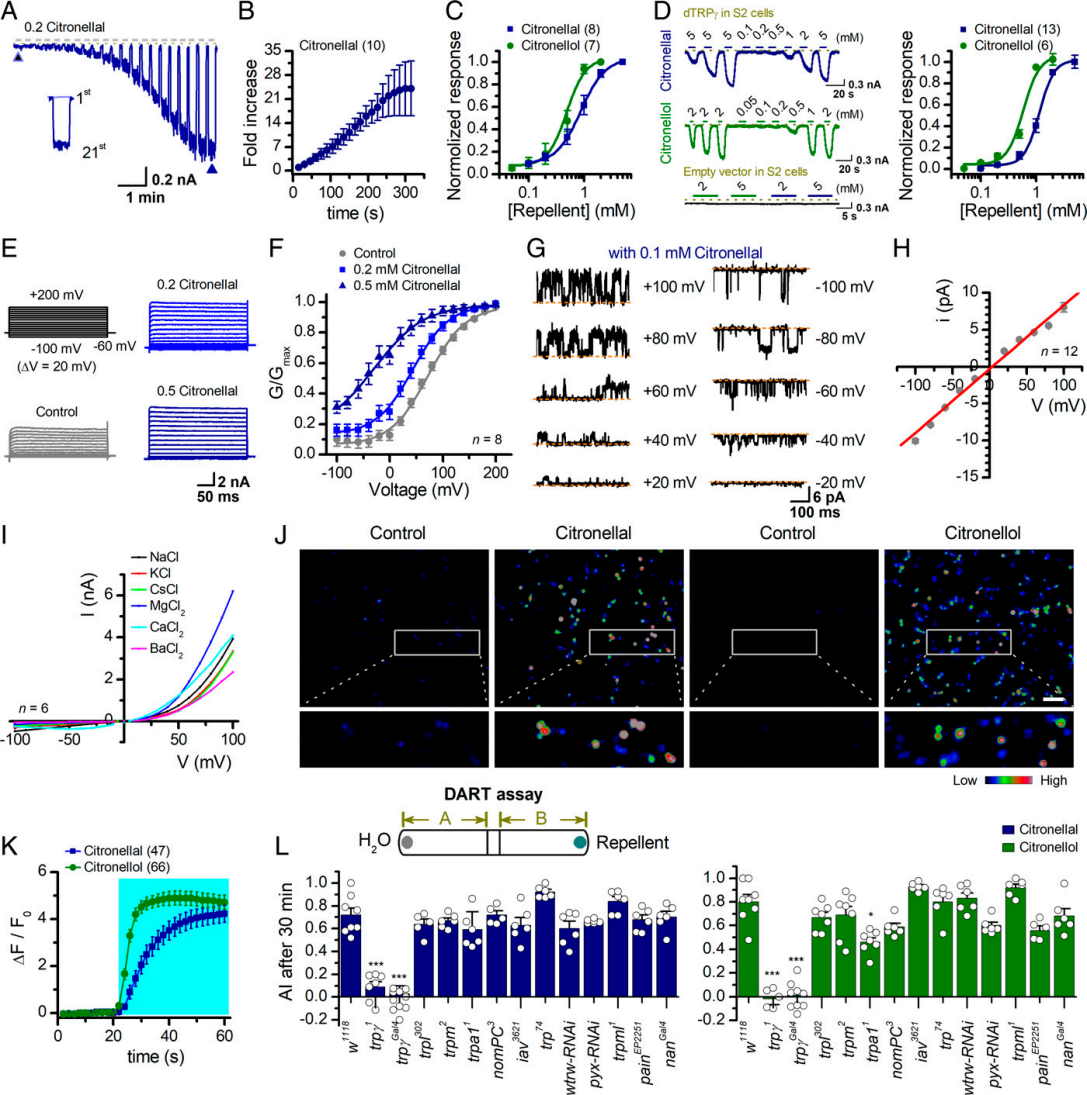
*Drosophila* TRPγ is necessary for the avoidance to natural repellents. (*A*) Representative whole-cell recordings of dTRPγ-expressing HEK 293T cells in response to repeated applications of 0.2 mM citronellal. A comparison between the responses to the first and the 21st applications is shown in *Inset*. The dotted line indicates zero current level. (*B*) Statistical plot of fold increase of peak response over repetitive stimulation. Data were normalized to the first pulse response (*n* = 10). (*C*) Dose-response curves of citronellal and citronellol for activation of dTRPγ channels. Solid lines indicate fits to the Hill equation, yielding EC_50_ = 0.87 ± 0.05 mM and n_H_ = 2.0 ± 0.2 for citronellal (*n* = 8), EC_50_ = 0.49 ± 0.02 mM and n_H_ = 2.5 ± 0.3 for citronellol (*n* = 7). (*D*) Representative traces of whole-cell recordings of *Drosophila* S2 cells transiently transfected with dTRPγ or empty vector in response to varying concentration of citronellol or citronellal after sensitization. (*Right*) Concentration-response curves of repellents for activation of dTRPγ channels. Solid lines indicate fits to the Hill equation with EC_50_ = 1.14 ± 0.07 mM, n_H_ = 3.3 ± 0.6 for citronellal (*n* = 13); and EC_50_ = 0.59 ± 0.05 mM, n_H_ = 3.0 ± 0.7 for citronellol (*n* = 6). (*E*) Representative whole-cell currents of dTRPγ-expressing HEK 293T cells elicited by a family of voltage pulses ranging from −100 mV to 200 mV with a 20-mV increment as indicated at upper left, in the presence of the bath solution (control), 0.2 mM citronellal, or 0.5 mM citronellal. V_h_ = -60 mV. (*F*) G-V relationships derived from the recordings shown in *E*. Solid lines correspond to fits with Boltzmann function, yielding V_1/2_ = 72.1 ± 2.6 mV for control; V_1/2_ = 43.3 ± 2.1 mV for 0.2 mM citronellal, and V_1/2_ = −29.7 ± 7.9 mV for 0.5 mM citronellal (*n* = 8). (*G*) Single-channel currents of dTRPγ recorded from outside-out membrane patches of HEK 293T cells evoked by 0.1 mM citronellal at the indicated holding potentials after sensitization induced by 2 mM citronellal. Dotted lines indicate the closed channel state. (*H*) Plot of unitary current amplitudes versus voltages. The unitary currents were determined by fitting all-point histograms with Gaussians. Unitary conductance assessed by fitting a linear function was 86.1 ± 0.5 pS (*n* = 12). (*I*) Current-voltage relations of dTRPγ in the presence of 0.5 mM citronellal, with bath solutions containing different cations as indicated. Pipette solutions contained 140 mM NaCl. Currents were elicited with 100-ms test pulses ranging from −100 mV to +100 mV with an increment of 10 mV (*n* = 6). (*J*) [Ca^2+^]_i_ increases elicited by different agonists. Responses of dTRPγ-expressing HEK 293T cells to 2 mM citronellal or 2 mM citronellol measured by GCaMP6m fluorescence with 1.8 mM extracellular Ca^2+^. Color bar indicates the calibration of intracellular calcium concentration, [Ca^2+^]_i_. Activation of dTRPγ resulted in the rise of [Ca^2+^]_i_. (Scale bar, 100 μm.) (*K*) Time courses of the relative changes of GCaMP6m fluorescence. (*L*) Summary of avoidance responses of *dTRP-KO* strains to 2 mM citronellal or 2 mM citronellol, with the schematic representation of the DART assay shown above. Error bars represent SEM. In the citronellal panel, ***P = 1.11E−4 for *trpγ^1^*, 4.85E−5 for *trpγ^Gal4^*; in the citronellol panel, ***P = 1.63E−5 for *trpγ^1^*, 7.55E−6 for *trpγ^Gal4^*, *P = 0.027 for *trpA1^1^* versus WT control by ANOVA.

Like sTRP1, dTRPγ activation by citronellal displayed step-wise sensitization upon repetitive stimulation (Fig. 4 *A* and *B*). The fully sensitized channel exhibited EC_50_ values of 0.87 ± 0.05 mM for citronellal (*n* = 8) and 0.49 ± 0.02 mM for citronellol (*n* = 7) (Fig. 4*C*). The expression of dTRPγ in *Drosophila* Schneider 2 (S2) cells also resulted in reconstitution of repellent-evoked currents, while S2 cells transfected with empty vector had no detectable response to the repellents (Fig. 4*D*), demonstrating their chemosensitivity in the *Drosophila* cell background. We also examined the voltage-dependent activation of dTRPγ under the control condition and in the presence of citronellal (Fig. 4*E*). Notably, the channel activity was small when evoked by voltage alone but increased in the presence of citronella. The G-V curves were individually fit to a Boltzmann equation with V_1/2_ values of 72.1 ± 2.6 mV for the control (*n* = 8), 43.3 ± 2.1 mV for 0.2 mM citronellal (*n* = 8), and −29.7 ± 7.9 mV for 0.5 mM citronellal (*n* = 7) (Fig. 4 *E* and *F*). Unitary currents were detected in excised patches at 0.1 mM citronellol, showing a linear I-V relationship and a slope conductance of 86.1 ± 0.5 pS (*n* = 12; Fig. 4 *G* and *H*). Ion selectivity analysis (Fig. 4*I* and *SI Appendix*, Fig. S8) confirmed that dTRPγ is more selective for divalent than monovalent cations. In addition, Ca^2+^ imaging showed that dTRPγ was able to mediate intracellular Ca^2+^ elevation in responses to citronellal or citronellol as sTRP1 (Fig. 4 *J* and *K*). Using RT-qPCR, we observed dTRPγ to be widely expressed throughout the body parts of the fruit fly (*SI Appendix*, Fig. S9).

We then validated the role of dTRPγ in mediating *Drosophila* avoidance behaviors to natural repellents. We conducted a two-choice preference assay similar to the direct airborne repellent test (DART) assay, but without screens (22), using the wild-type (WT) *w^1118^* flies and multiple *dTRP-knockout* (*KO*) strains (23). We found that the *w^1118^* flies displayed strong avoidance to citronellal and citronellol (Fig. 4*L*). Confirming the critical and specific role of dTRPγ in chemosensing, the chemorepulsion to citronellal and citronellol was impaired in *dtrpγ* mutants, and the behavioral phenotype was indistinguishable between *dtrpγ^Gal4^* and *dtrpγ^1^*. Furthermore, we conducted calcium imaging in the antenna of the *Drosophila* olfactory receptor organs. We constructed two *Drosophila* strains by expressing the fluorescent calcium indicator GCaMP6 driven by *dtrpγ-Gal4* either in the WT strain (*trpγ-Gal4/UAS-GCaMP*) or dTRPγ-KO strain (*trpγ-Gal4/trpγ^1^; UAS-GCaMP*). We observed that Ca^2+^ levels were remarkably elevated in the WT antenna when challenged with citronellal or citronellol, while no increase was detected in the KO strains (*SI Appendix*, Fig. S10). Thus, as the sTRP1 ortholog, dTRPγ also functions as a receptor for natural repellents, exemplifying them as arthropod molecular targets of plant-derived repellents.

### Molecular Determinants of sTRP1/dTRPγ Activation.

Next, we sought to explore key residues involved in sTRP1/dTRPγ activation by plant-derived repellents. As both sTRP1 and dTRPγ channels belong to the TRPC subfamily, we selected the human (h) TRPC3 channel for chimeric analysis. As illustrated in *SI Appendix*, Fig. S11*A*, whole-cell recording in transiently transfected HEK 293T cells revealed that hTRPC3 channels were robustly activated by 1-oleoyl-2-acetyl-snglycerol (24) but showed no response to citronellal or citronellol. Using hTRPC3 as an orthogonal template, we constructed a series of chimeric channels by replacing each of the transmembrane linker domains of sTRP1 with the cognate region of hTRPC3 (*SI Appendix*, Fig. S11*B*). The function of the chimeric channels was tested by whole-cell recording in transiently transfected HEK 293T cells. The replacement of the N terminus, S4-S5 linker (L45), and Ct of sTRP1 rendered them insensitive to the natural repellents, and chimera sTRP1/C3(L34) also retained little activity (*SI Appendix*, Fig. S11 *B* and *C*), implying that residues within these domains contribute to repellent activation of sTRP1.

Functional and structural data have documented the cytosolic L45 as a gearbox in TRP channel gating (25). For instance, structural analysis of TRPV1 protein has identified the L45 region as an important binding pocket for vanilloid and PI(4,5)P_2_ (26). We then made single amino acid substitutions within the L45 of sTRP1, based on its alignment with hTRPC3. While the M483Y substitution of sTRP1 displayed a low response to camphor, it did not alter the response to citronellal and citronellol. The Y485L and S489E substitutions significantly reduced the sensitivity to citronellal but retained the robust response to citronellol (*SI Appendix*, Fig. S11 *D* and *E*). Using the same strategy, we also screened nine different residues in dTRPγ (*SI Appendix*, Fig. S11*B*). We found that the V515A substitution selectively impaired the sensitivity to citronellol and camphor, mutant V513L reduced the response to citronellal and citronellol, and K508R exhibited reduced sensitivities to camphor (*SI Appendix*, Fig. S11 *F* and *G*). Further, we found that the mutation dTRPγ(H518A), also located in the L45, selectively lost the response to citronellal (*SI Appendix*, Fig. S12*A*). Accordingly, reconstitution of WT TRPγ in *trpγ^1^* flies could restore the response to both citronellal and citronellol, while reintroducing TRPγ(H518A) into *trpγ^1^* flies could only rescue the avoidance response to citronellal (*SI Appendix*, Fig. S12*B*). Together, these results support the involvement of the L45 domain in the responses of sTRP1 and dTRPγ to the natural repellents.

## Discussion

By gene library screening in *M. martensii*, we identified sTRP1 as a molecular target of natural repellents. This receptor shows high to moderate sensitivity to citronellal, citronellol, and camphor, the plant-derived insect repellents. In contrast, sTRP1 is insensitive to the synthetic chemical pesticide DEET. We observed that DEET could cause avoidance behaviors in *M. martensii*. Genetic and physiological studies conducted in *Anopheles gambiae*, *D. melanogaster*, and *Culex quinquefasciatus* suggested that the molecular receptors for DEET belong to the odorant receptors and gustatory receptors (27–30). Likely, such signal pathways might function in *M. martensii* scorpions to regulate their response to DEET.

The identification of sTRP1 also enabled the determination of a function for dTRPγ, a TRPC channel found in *Drosophila* and previously implicated in insect light sensing (31), fine motor control (23), and olfaction (32). However, its gating property and ion channel function have not been fully defined (23). Our data demonstrate that both dTRPγ and sTRP1 are directly activated by the natural repellents to initiate avoidance behavior. Sequence mapping suggests that both sTRP1 and dTRPγ belong to the TRPC subfamily, which is well retained among species (*SI Appendix*, Fig. S3). Interestingly, another TRPC family channel, dTRPL, was reported to be a taste sensor in gustatory receptor neurons in *Drosophila*, mediating their aversion to a camphor diet (33). This channel was found dispensable for the repelling effect of camphor, thus excluding its role in fly olfactory sensing (33). We here show that camphor activates both sTRP1 and dTRPγ (*SI Appendix*, Fig. S11), which hence would also contribute to the avoidance behaviors to camphor. The ion channel TRPA1 and its variants have also been suggested to be involved in insect sensing of natural repellents (22, 34–37). Our current data show that the *trpa1^1^* mutant flies showed only modest effects on their avoidance responses to citronellal and citronellol (Fig. 4*L*). This observation likely reflects the indirect participation of dTRPA1 in chemosensation that is mediated by G protein/phospholipase C signaling cascade as previously reported (22). In addition, two *Drosophila* TRPV channels, Nanchung and Inactive, were reported to be sensitive to synthetic chemical insecticides Pymetrozine and Pyrifluquinazon, respectively (38). Hence, our study not only pinpoints sTRP1 and dTRPγ as direct molecular targets of natural repellents, but it also delineates the functional specialization of TRP ion channels in arthropod sensing of chemical environments.

*M. martensii* possesses an ancestral and highly preserved genome over arthropod evolution. Genomic analysis indicates that TRP genes in insects are also well conserved, suggesting the high evolutionary conservation of the mechanisms for integrating environmental signals (39). The insect-repelling plants (e.g., *Cymbopogon*, from which the citronellal and citronellol are derived) are naturally distributed in environments. The initial low-level reaction of the TRP ion channels to those plants would allow the insects to hunt or pass over the plants transiently. Hence, this gradual sensation process of sTRP1 and dTRPγ likely represents an evolutionary adaptation to the natural environments for better survival. We detected a wide expression of the sTRP1 genes throughout the scorpion body, while their tissue distribution at the protein level remains to be examined due to the current lack of suitable antibodies. Whether the chemosensing behavior mediated by sTRP1 is linked to smell, taste, or skin or tissue contact in scorpions needs further exploration. We show that the L45 region is crucial for sTRP1/dTRPγ activation, thereby providing a mechanistic ground for understanding their interaction with the natural repellents.

Our study demonstrates a molecular basis underlying the sensing of arthropods to plant-derived natural repellents, thereby advancing our understanding of the insect chemosensory principles to pesticides and ecosystem homeostasis (40, 41). This should also help the rational design of ecological pest control strategies while preserving biodiversity.

## Materials and Methods

### Avoidance Response Assay.

The avoidance response assay in scorpion and *Drosophila* was performed using a similar protocol as described to examine the two-choice preference of mice or *Drosophila* (22). For scorpion avoidance responses, there were two pieces of gauze (8 cm × 8 cm) preimmersed with 2 mL water (control) or drugs (citronellol 50% vol/vol; citronellal 50% vol/vol; DEET 100%; menthol 500 mM; and camphor 500 mM) placed on one side of a container (100 cm × 50 cm × 30 cm) with a layer of clean sand, and each gauze was covered by a piece of tile (18 cm × 12 cm). For each test, 100 fresh scorpions were initially placed at the center of the container covered with a piece of glass (100 cm × 50 cm). The test scorpions were allowed to freely crawl, and about 1 h later, the numbers of scorpions under and outside the tiles were counted separately. Sand and tiles were replaced with new materials, and the container was washed thoroughly after each trial.

A similar strategy was used for the avoidance assay in *Drosophila*. Two 15-mL test tubes were prepared and allowed to open to the air for more than 24 h to remove residual chemical odors. The drugs were applied to a piece of Kimwipe (∼10 mm × 10 mm) and placed at the bottom of the tube. Then the tubes were attached using a three-way connector. For each trial, 50 untested flies were gently tapped into the tube through the connector. The outlet was sealed by a plug. The tested tubes were then placed at a 25 °C incubator. After 30 min, the number of flies in each test tube was counted separately, and the avoidance index (AI) was calculated using the equation AI = (# in A − # in B)/(# in A + # in B), where “# in A” and “# in B” are the number of flies in control tube A and experimental tube B, respectively. For statistical analysis, six to eight independent trials were performed.

### cDNA Constructs and Mutagenesis.

Total scorpion RNA was extracted from *M. martensii* using RNAiso reagent (Takara Biotechnology Co., Dalian, China) following the manufacturer’s protocol. RT-PCR was performed with the RevertAid First Strand cDNA Synthesis Kit (Thermo, USA) to generate the cDNA library. The full-length cDNAs for *strp1–6* were cloned by RACE PCR from the *M. martensii* cDNA library and subcloned into pIRES2-EGFP vector. The primers designed for cloning s*trp1–6* are summarized in *SI Appendix*, Table S2. Human TRPC3 was gifted from Wei Yang, Zhejiang University, Hangzhou, Zhejiang Province, China. The *Drosophila* NompC channel was generously provided by Wei Zhang, Tsinghua University, Beijing, China, and Zhiqiang Yan, Fudan University, Shanghai, China, and the full-length cDNAs of 12 *Drosophila* TRP channels as indicated were obtained from *D. melanogaster* cDNA library and subcloned into the pIRES2-EGFP vector.

### Cell Culture and Expression.

Scorpion sensory neurons were dissociated from VNC ganglia of *M. martensii*. Briefly, the adult scorpions were decapitated, and the VNC ganglia were exposed simply by removing the carapace, tergites, dorsal surface of the postabdominal rings, dorsal surface of the telson, and tissue adjacent to the central nervous system. Thereafter, three pairs of preabdominal VNC ganglia were immediately dissected and rinsed in Ca^2+^/Mg^2+^-free Hank’s balanced salt solution. Ganglia were dissociated by enzymatic treatment with collagenase (Type IA, 1 mg/mL) and trypsin (type I, 0.3 mg/mL) at 30 °C for 30 min. During digestion, gentle mechanical trituration was performed every 10 min through fire-polished glass pipettes until solution become cloudy. The resulting suspension of single cells was centrifuged at 1,500 rpm for 5 min and resuspended in Dulbecco’s modified Eagle’s medium containing 10% heat-inactivated fetal bovine serum, then seeded onto a poly-L-lysine pretreated glass slides and cultured in a humidified incubator gassed with 5% CO_2_. Electrophysiology experiments were performed ∼4 to 8 h after the plating.

### Other Materials and Methods.

Details for the cell culture, expression, electrophysiology, and other methods described are provided in *SI Appendix*, *SI Materials and Methods*.

## Supplementary Material

Supplementary File

Supplementary File

## Data Availability

Plasmids data and all datasets supporting the conclusions of this article have been deposited in Dryad (https://doi.org/10.5061/dryad.nk98sf7vp) (42). All other study data are included in the article and/or supporting information.
