# Supplementary material for: Identification of an arthropod molecular target for plant-derived natural repellents
Source: Proc Natl Acad Sci U S A. 2022 Apr 22;119(18):e2118152119. doi: 10.1073/pnas.2118152119 (PMC9170154; doi:10.1073/pnas.2118152119)
Supplement: Supplementary File [file pnas.2118152119.sapp.pdf]

PNAS

Supplementary Information for

Identification of an arthropod molecular target for plant-derived natural repellents

Quan Tian[a,b,c,d,1], Peiyu Wang[a,b,c,d,1], Chang Xie[a,b,c,d,1], Peiyuan Pang[a,b,c,d], Youjing Zhang[a,b,c,d], Yue Gao[a,b,c,d], Zhijian Cao[a,e], Yingliang Wu[a,e], Wenxin Li[a,e], Michael X. Zhu[f], Dongdong Li[g], Jing Yao [a,b,c,d,2]

[a]State Key Laboratory of Virology, College of Life Sciences, Wuhan University, Wuhan, Hubei 430072, China.

[b]Hubei Key Laboratory of Cell Homeostasis, College of Life Sciences, Wuhan University, Wuhan, Hubei 430072, China.

[c]Department of Anesthesiology, Zhongnan Hospital of Wuhan University, Wuhan, Hubei 430072, China.

[d]Frontier Science Center for Immunology and Metabolism, Wuhan University, Wuhan, Hubei 430072, China.

[e]Modern Virology Research Center, College of Life Sciences, Wuhan University, Wuhan, Hubei 430072, China.

[f]Department of Integrative Biology and Pharmacology, McGovern Medical School, The University of Texas Health Science Center at Houston, Houston, TX 77030.

[g]Sorbonne Université, Institute of Biology Paris Seine, Neuroscience Paris Seine, CNRS UMR8246, INSERM U1130, Paris 75005, France.

[1]These authors contributed equally to this work

[2]Corresponding author. Email: jyao@whu.edu.cn

This PDF file includes:

SI materials and methods
SI references
Figures S1 to S12
Tables S1 to S4
Legends for Movies S1

Other supplementary materials for this manuscript include the following:

Movies S1

SI Materials and Methods

Experimental Animals

Mesobuthus Martensii scorpions were collected from the Fu-niu Mountains, Xichuan County, Henan province, China. *Drosophila* w^{1118} was used as the wild-type control. All *Drosophila* mutants as listed in Supplementary Table 1 were purchased from Bloomington Stock Center. The *UAS-trpγ (WT)*, *UAS-trpγ (H518A)* and *UAS-GCaMP Drosophila* strains were generated by Core Facility of Drosophila Resource and Technology, CEMCS, CAS in Shanghai, China. The full length cDNAs were subcloned into the pUAST-attB vector. The recombined vectors were then injected into the w^{1118} embryos and the transformants were identified based on w^{+} eye color.

Avoidance Response assay

The avoidance response assay in scorpion and *Drosophila* was performed using a similar protocol as described to examine the two-choice preference of mice or *Drosophila* (1). For scorpion avoidance responses, there were two pieces of gauze (8 cm × 8 cm) pre-immersed with 2 ml water (control) or drugs (citronellol 50% vol/vol; citronellal 50% vol/vol; DEET 100%; menthol 500 mM; and camphor 500 mM) placed on one side of a container (100 cm × 50 cm × 30 cm) with a layer of clean sand, and each gauze was covered by a piece of tile (18 cm × 12 cm). For each test, 100 fresh scorpions were initially placed at the center of the container covered with a piece of glass (100 cm × 50 cm). The test scorpions were allowed to freely crawl, and about 1 h later, the numbers of scorpions under and outside the tiles were counted separately. Sand and tiles were replaced with new materials and container was washed thoroughly after each trial.

A similar strategy was used for the avoidance assay in *Drosophila*. Two 15-ml test tubes were prepared and allowed to open to the air for more than 24 h to remove residual chemical odors. The drugs were applied to a piece of Kimwipe (~10 mm × 10 mm) and placed at the bottom of the tube. Then the tubes were attached together using a three-way connector. For each trial, 50 untested flies were gently tapped into the tube through the connector. The outlet was sealed by a plug. The tested tubes were then placed at a 25°C incubator. After 30 min, the number of flies in each test tube was counted separately and the avoidance index (AI) was calculated using the equation: AI = (# in A - # in B) / (# in A + # in B), where # in A and # in B are the number of flies in control tube A and experimental tube B, respectively. For statistical analysis, 6-8 independent trials were performed.

cDNA constructs and mutagenesis

Total scorpion RNA was extracted from *M. martensii* using RNAiso reagent (Takara Biotechnology Co., Dalian, China) following the manufacturers' protocol. RT-PCR was performed with RevertAid First Strand cDNA Synthesis Kit (Thermo, USA) to generate cDNA library. The full-length cDNAs for *sTRP1-6* were cloned by rapid amplification of cDNA ends (RACE) PCR from the *M. martensii* cDNA library and subcloned into pIRES2-EGFP vector. The accession numbers for each sequence in GeneBank as following: sTRP1, OM777143; sTRP2, OM777144; sTRP3, OM777145; sTRP4, OM777146; sTRP5, OM777147; sTRP6, OM777148. The primers designed for cloning s*TRP1-6* are summarized in Supplementary Table 2. Human TRPC3 was gifted

from Dr. Wei Yang (Zhejiang University, Hangzhou, Zhejiang, China). *Drosophila* NompC channel was generously provided by Dr. Wei Zhang (Tsinghua University, Beijing, China) and Zhiqiang Yan (Fudan University, Shanghai, China), and the full-length cDNAs of 12 *Drosophila* TRP channels as indicated were obtained from *D. melanogaster* cDNA library and subcloned into the pIRES2-EGFP vector. Chimeras and site-directed mutagenesis were performed using the overlap-extension polymerase chain reaction (PCR) method as previously described(2). To express d*TRPγ* in *Drosophila* Schneider 2 (S2) cells, the full-length cDNA of d*TRPγ* was constructed into pAc5.1/V5-His vector. All recombinant constructs were verified by DNA sequencing.

For the analysis of expression patterns of the gene, the body of scorpion was anatomically divided into 5 segments: pedipale, prosoma, mesasoma, metasoma and ambulatory legs. Similarly, the body of *Drosophila* was also divided into 5 segments: proboscis, head, thorax, abdomen, and legs. Separated body parts were then treated with TriZol (Invitrogen) to obtain RNA. Afterwards, the first-strand cDNA was synthesized with a reverse transcription kit (Invitrogen). Gene expression was examined with a Bio-Rad SFX connect system using Bestar qPCR Master Mix (DBI, Germany) (SYBR Green). Data were normalized to the expression of β-Actin. Gene specific primers were shown in Supplementary Table 3.

Cell culture and expression

Scorpion sensory neurons were dissociated from ventral nerve cord (VNC) ganglia of *Mesobuthus Martensii*. Briefly, the adult scorpions were decapitated, and the VNC ganglia were exposed simply by removing the carapace, tergites, dorsal surface of the postabdominal rings, dorsal surface of the telson, and tissue adjacent to the central nervous system. Thereafter, three pairs of preabdominal VNC ganglia were immediately dissected and rinsed in Ca^{2+}/Mg^{2+}-free Hank's balanced salt solution. Ganglia were dissociated by enzymatic treatment with collagenase (Type IA, 1 mg/ml) and trypsin (type I, 0.3 mg/ml) at 30 °C for 30 min. During digestion, gentle mechanical trituration was performed every 10 min through fire-polished glass pipettes until solution become cloudy. The resulting suspension of single cells was centrifuged at 1,500 rpm for 5 minutes, and resuspended in Dulbecco's modified Eagle's medium (DMEM, Thermo Fisher Scientific, MA) containing 10% heat-inactivated fetal bovine serum (FBS), then seeded onto a poly-L-lysine pre-treated glass slides, and cultured in a humidified incubator gassed with 5% CO_2. Electrophysiology experiments were performed ~4-8 h after the plating.

HEK 293T cells were grown in DMEM containing 4.5 mg/ml glucose, 10% FBS, 50 units/ml penicillin, and 50 μg/ml streptomycin, and were incubated at 37°C in a humidified incubator gassed with 5% CO_2. *Drosophila* S2 cells were kindly gifted from Dr. Xi Zhou (Wuhan Institute of Virology, Chinese Academy of Sciences). S2 cell were grown in Schneider's Insect Medium (Millipore Sigma, St. Louis, MO) supplemented with 10% heat-inactivated FBS, and were incubated at 27 °C in a precision biochemical incubator. Cells at a confluence of ~70% were transfected with the desired DNA

constructs using Lipofectamine 2000 (Invitrogen, CA, USA) according to the manufacturer's instructions, or using a standard calcium phosphate precipitation method. Transfected cells were reseeded on 12 mm round glass coverslips coated by poly-L-lysine. Experiments took place ~48 h after transfection.

Electrophysiology

Conventional whole-cell and excised patch-clamp recording methods were used. Cells were voltage clamped or current clamped in the whole-cell mode using an EPC10 amplifier (HEKA, Lambrecht, Germany). Voltage commands were made from the Patchmaster program. For a subset of recordings, currents were amplified by an Axopatch 200B amplifier (Molecular Devices, Sunnyvale, CA) and recorded through a BNC-2090/MIO acquisition system (National Instruments, Austin, TX) using QStudio developed by Dr. Feng Qin at the State University of New York at Buffalo. Recording pipettes were pulled from borosilicate glass capillaries (World Precision Instruments, WPI), and fire-polished to a resistance of 2-4 MΩ when filled with internal solution. Whole-cell recordings were typically sampled at 5 kHz and filtered at 1 kHz, and single-channel recordings were sampled at 25 kHz and filtered at 10 kHz.

Electrodes were filled with (in mM): 150 CsCl, 5 EGTA, 10 HEPES, pH 7.4 adjusted with CsOH. The control bath solution contained (in mM): 140 NaCl, 5 KCl, 2 $CaCl_2$, 10 HEPES, pH 7.4 adjusted with NaOH. The Ca^{2+}-free bath saline for whole-cell recording in HEK 293T cells consisted of (in mM): 140 NaCl, 5 KCl, 3 EGTA, and 10 HEPES, pH 7.4 adjusted with NaOH. For recordings in ventral nerve cord neurons, the bath solution consisted of (in mM): 140 NaCl, 5 KCl, 2 $MgCl_2$, 2 $CaCl_2$, 10 glucose, 10 HEPES, pH 7.4 adjusted with NaOH. The bath and pipette solutions for single-channel recording was symmetrical and contained 140 NaCl, 5 KCl, 5 EGTA, and 10 HEPES, pH 7.4 (adjusted with NaOH). For cation substitution experiment, normal bath solution was used to establish a whole-cell configuration. Pipette solution contains (in mM): 140 NaCl and 10 HEPES, pH 7.4 adjusted with NaOH. After the whole-cell configuration was obtained, bath solution was replaced with specific cationic solution, and a voltage step pulse was used to measure the reversal potential. For monovalent cation X^+ (Na^+, K^+, and Cs^+), cationic solution contains (in mM): 140 XCl, 10 HEPES, pH 7.4 adjusted with XOH. For divalent cation Y^{2+} (Mg^{2+}, Ca^{2+}, and Ba^{2+}), cationic solution contains (in mM): 110 YCl_2, 10 HEPES, pH 7.4 adjusted with $Y(OH)_2$. Isolated cells were voltage clamped and held at -60 mV before recordings. Exchange of external solutions was performed using a gravity-driven local perfusion system. As determined by the conductance tests, the solution around a patch under study was fully controlled by the application of a solution with a flow rate of 100 μl/min or greater. All pharmacological experiments met this criterion. All patch-clamp recordings were made at room temperature (22-24 °C).

Intracellular Ca^{2+} measurements

For intracellular Ca^{2+} measurements, cells were transfected with the desired DNA constructs together with GCaMP6m (gift from Dr. Liangyi Chen at Peking University). After transfection, cells were reseeded on a poly-L-lysine pre-treated glass coverslips. The bath solution consisted of (in mM): 140 NaCl, 5 KCl, 2 $MgCl_2$, 2 $CaCl_2$, 10 glucose, 10 HEPES, pH 7.4 adjusted with NaOH. In another set of experiment, fly antennas were acutely dissected in bath solution consisted of (in mM): 140 NaCl, 5 KCl, 2 $MgCl_2$, 2 $CaCl_2$, 10 glucose, 10 HEPES, pH 7.15 adjusted with NaOH and fixed on a silicone (MOLYKOTE) pre-treated glass dish. Calcium imaging was performed on an inverted epifluorescence microscope (Olympus IX 73) equipped with a complete illumination system (Lambda XL, Sutter Instruments). Fluorescent images were acquired using a cool CCD camera (CoolSNAP ES2, Teledyne Photometrics) controlled by Micro-Manager 1.4 (Vale lab, UCSF) using a public 1394 digital camera driver (Carnegie Mellon University). Images at excitation of 470 nm and emission of 510 nm were taken at 0.2 or 1 Hz.

Immunostaining

HEK 293T cells transfected with desired DNA constructs were prepared for immunostaining, which took place 16-24 hours after transfection. Samples were washed twice with phosphate-buffered saline (PBS), and then were fixed with ice-cold methanol for 10 min, and thereafter blocked in PBS supplemented with 5% FBS for 30 min. Afterwards, samples were incubated with rabbit monoclonal anti-human Na^+/K^+-ATPase antibody at a dilution of 1:500 in 1% BSA for 1 h, and were then incubated with conjugated antibodies, the donkey anti-rabbit IgG-FITC at a dilution of 1:1000 in 1% BSA for 1 h. Then cells were seeded on glass slides with DAPI. All the procedures were performed at room temperature (22-25 °C). The outcomes were evaluated by a confocal microscopy.

Antibodies and drugs

Rabbit monoclonal anti-human Na^+/K^+-ATPase antibody was purchased from Abcam (1:500, ab76020, RRID: ab76020; Cambridge, MA, USA), and donkey anti-rabbit IgG-FITC was purchased from Dakewe Biotech (1:500, 406403, RRID: ab893531; Shenzhen, China). Citronellal, citronellol were purchased from Aladdin (Aladdin, Shanghai, China). Unless otherwise noted, all other chemicals were ordered from Sigma (Millipore Sigma, St. Louis, MO). Citronellal, citronellol, 1-Oleoyl-2-acetyl-sn-glycerol (OAG), ruthenium red (RR) and 2-aminoethyl diphenylborinate (2-APB) were dissolved in dimethylsulfoxide (DMSO) or 100% ethanol to make a stock solution. All the stocks were diluted in the bath solution to the desired concentrations right before the experiment. The final concentrations of ethanol or DMSO did not exceed 0.3%, which had no effect on the currents.

Phylogenetic analysis

TRP channels of some representative species used for the analysis were shown in Supplementary Table 4. The alignments were performed using ClustalX (version 1.83), and phylogenetic trees were built with MEGA7 using Neighbor-Joining method (1,000 replicates for the bootstrapping tests).

Data analysis

Data were analyzed offline with Clampfit (Molecular Devices, Sunnyvale, CA), IGOR (Wavemetrics, Lake Oswego, OR, USA), SigmaPlot (SPSS Science, Chicago, IL) and OriginPro (OriginLab Corporation, MA, USA). For concentration dependence analysis, the modified Hill equation was used: $Y = A1 + (A2 - A1) / (1 + (EC_{50}/[toxin])^{nH})$, in which EC_{50} is the half maximal effective concentration, and n_H is the Hill coefficient. Permeation ratios for monovalent cations to Na^+ (P_x/P_{Na}) were calculated using a modified GHK equation: $P_X/P_{Na} = \exp(\Delta V_{rev}F/RT)$, where V_{rev} is the reversal potential, F is Faraday's constant, R is the universal gas constant, and T is the absolute temperature. For measurements of divalent cations' permeability (P_Y/P_{Na}), the following equation was used: $P_Y/P_{Na} = [Na^+]_i \times \exp(\Delta VrevF/RT) \times (1+ \exp(\Delta VrevF/RT))/4[Y^{2+}]_o$, where the bracketed terms represent activities. Assumed activity coefficients are 0.75 for monovalents and 0.25 for divalents, following the methods established by Soledad Valera *et al*(3). Unless stated otherwise, the data are presented as the mean ± standard error (SEM), from a population of cells (n), with statistical significance assessed by Student's t test for two group comparison or One-way ANOVA test with Dunnett's method and One-way ANOVA with the Tukey-Kramer test was used for comparing multiple group comparisons. Significant difference is indicated by a p value less than 0.05 ($*p < 0.05$, $**p < 0.01$, and $***p < 0.001$).

SI Reference

1. Y. Kwon *et al.*, Drosophila TRPA1 Channel Is Required to Avoid the Naturally Occurring Insect Repellent Citronellal. *Current Biology* **20**, 1672-1678 (2010).
2. Q. Tian *et al.*, Recovery from tachyphylaxis of TRPV1 coincides with recycling to the surface membrane. *Proc Natl Acad Sci U S A* **116**, 5170-5175 (2019).
3. S. Valera *et al.*, A new class of ligand-gated ion channel defined by P2x receptor for extracellular ATP. *Nature* **371**, 516-519 (1994).

Supplementary Figure 1

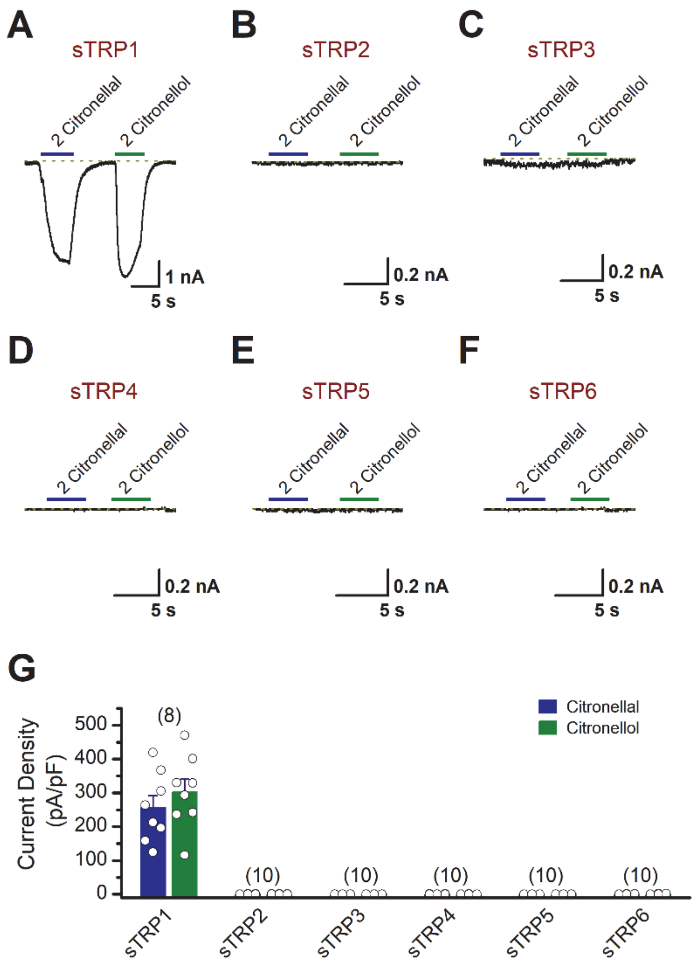

Figure S1. Of six scorpion TRP channels, only TRP1 responds to citronellal and citronellol.

(**A-F**) Representative whole-cell recordings from HEK293T cells expressing each potential TRP channel gene cloned from cDNA library of scorpions *M. Martensii*, in response to 2 mM citronellal and 2 mM citronellol, respectively. Holding potential was -60 mV. The dotted line indicates zero current level. (**G**) Summary of current densities of six different scorpion TRP channels evoked by 2 mM citronellal and 2 mM citronellol as shown in (**A-F**).

Supplementary Figure 2

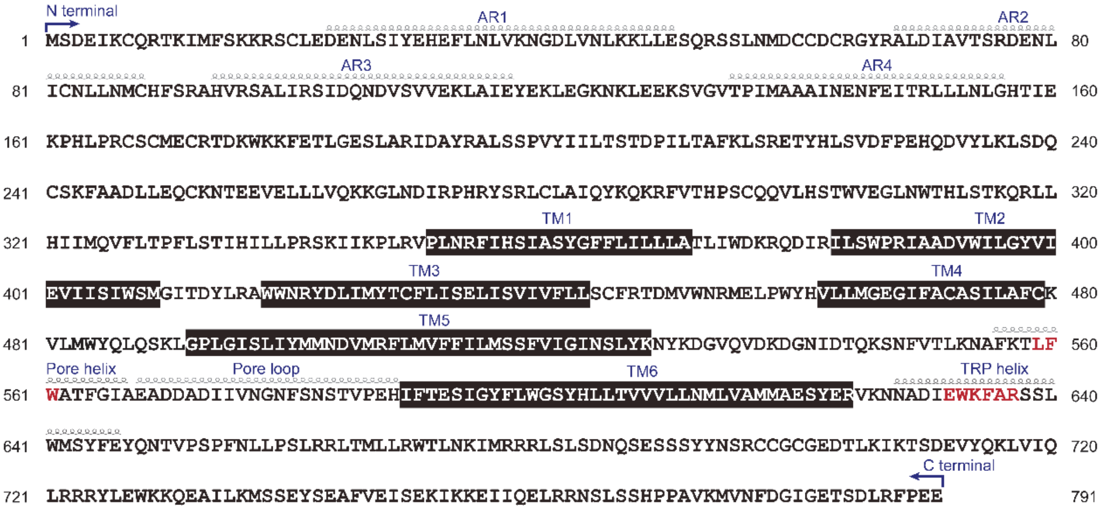

Figure S2. Predicted amino-acid sequence encoded by scorpion *trp1* gene and membrane topology of sTRP1 channel.

Amino acid sequence of full length sTRP1. Putative transmembrane domains (TM) are shaded in black, both the highly conserved LFW motif and TRP box region are in red. Spiral lines indicate putative ankyrin repeat domains (AR), pore helix, pore loop, and TRP helix regions, respectively.

Supplementary Figure 3

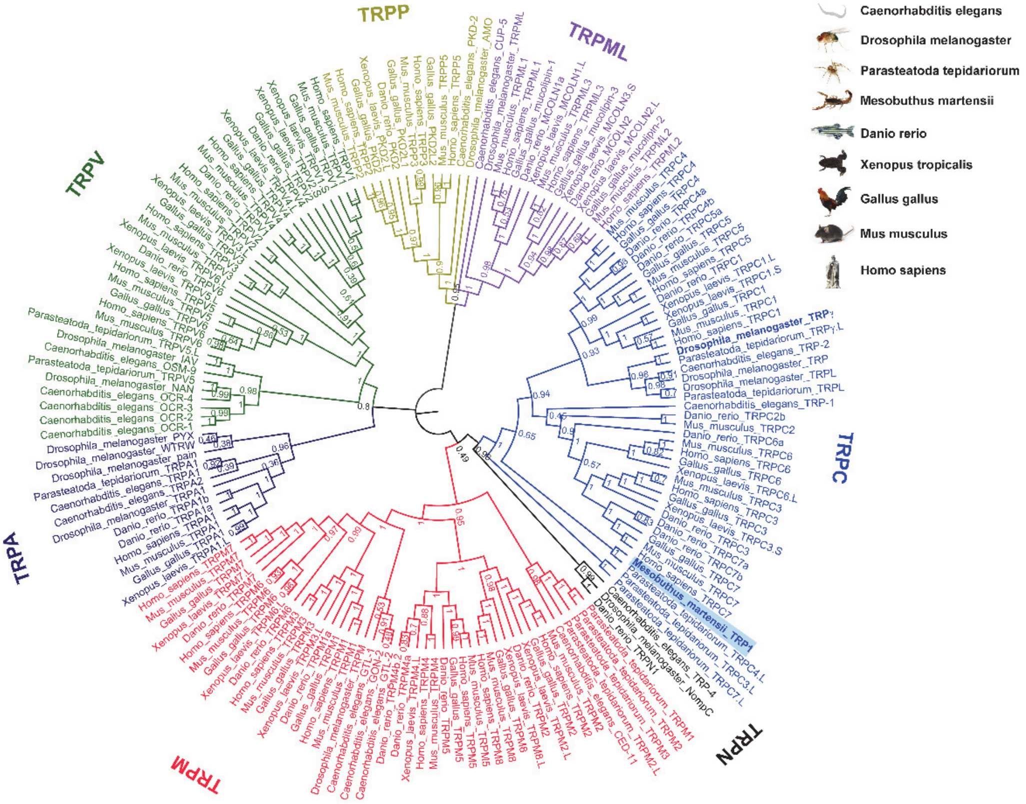

Figure S3. Phylogenetic relationship of the TRP channels.

Phylogenetic tree of TRP channels. Statistical confidence (bootstrap value) is indicated beside the respective branch. The tree was constructed using MEGA and ClustalX by comparing the amino acid sequences of TRP channels and was further built using Neighbor-joining method (1,000 replicates for the bootstrapping tests). sTRP1 is shaded in blue box.

Supplementary Figure 4

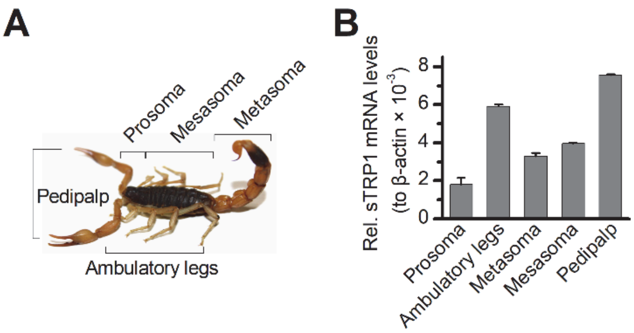

Figure S4. Expression patterns of scorpion *trp1* gene.

(**A**) Schematic of each body part of scorpion *Mesobuthus Martensii*. (**B**) Quantitative analysis of the relative mRNA levels (normalized by 10^{-3} fold of β-actin) of sTRP1 in the different parts of scorpion.

Supplementary Figure 5

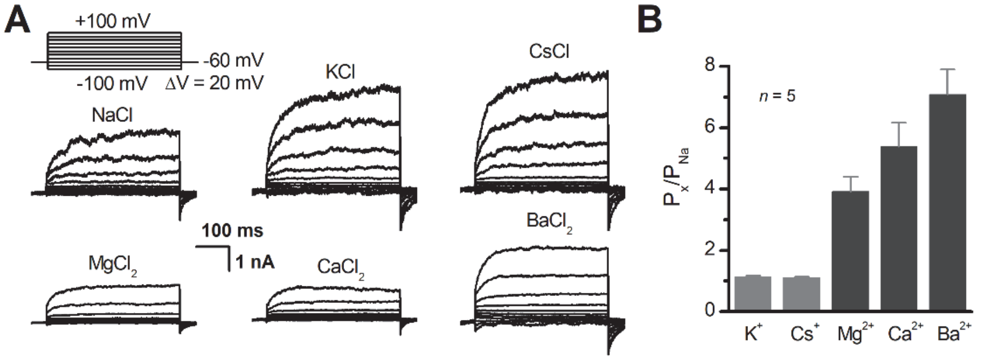

Figure S5. Ionic selectivity of sTRP1 channel.
(**A**) Representative whole-cell currents of HEK 293T cells expressing sTRP1 channels elicited by a family of voltage pulses ranging from -100 mV to 100 mV with a 20-mV increment in the presence of 0.5 mM citronellal, with bath solution containing different cations as indicated. Pipette solutions contained 140 mM NaCl. (**B**) Permeability ratios (P_X/P_{Na}) calculated from reversal potential shifts in (**A**).

Supplementary Figure 6

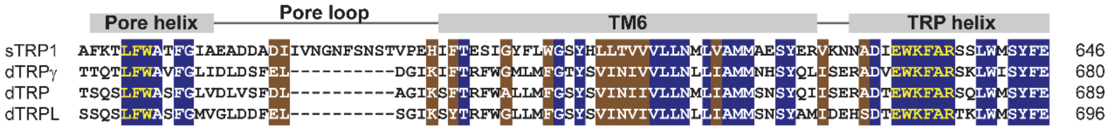

Figure S6. Alignment of the conserved regions among sTRP1, dTRPγ, dTRP and dTRPL channels.

Residues that are identical or similar among the sequences were shaded in blue and brown, respectively. Pore helix, pore loop, transmembrane domains 6(TM6), and TRP helix are indicated.

Supplementary Figure 7

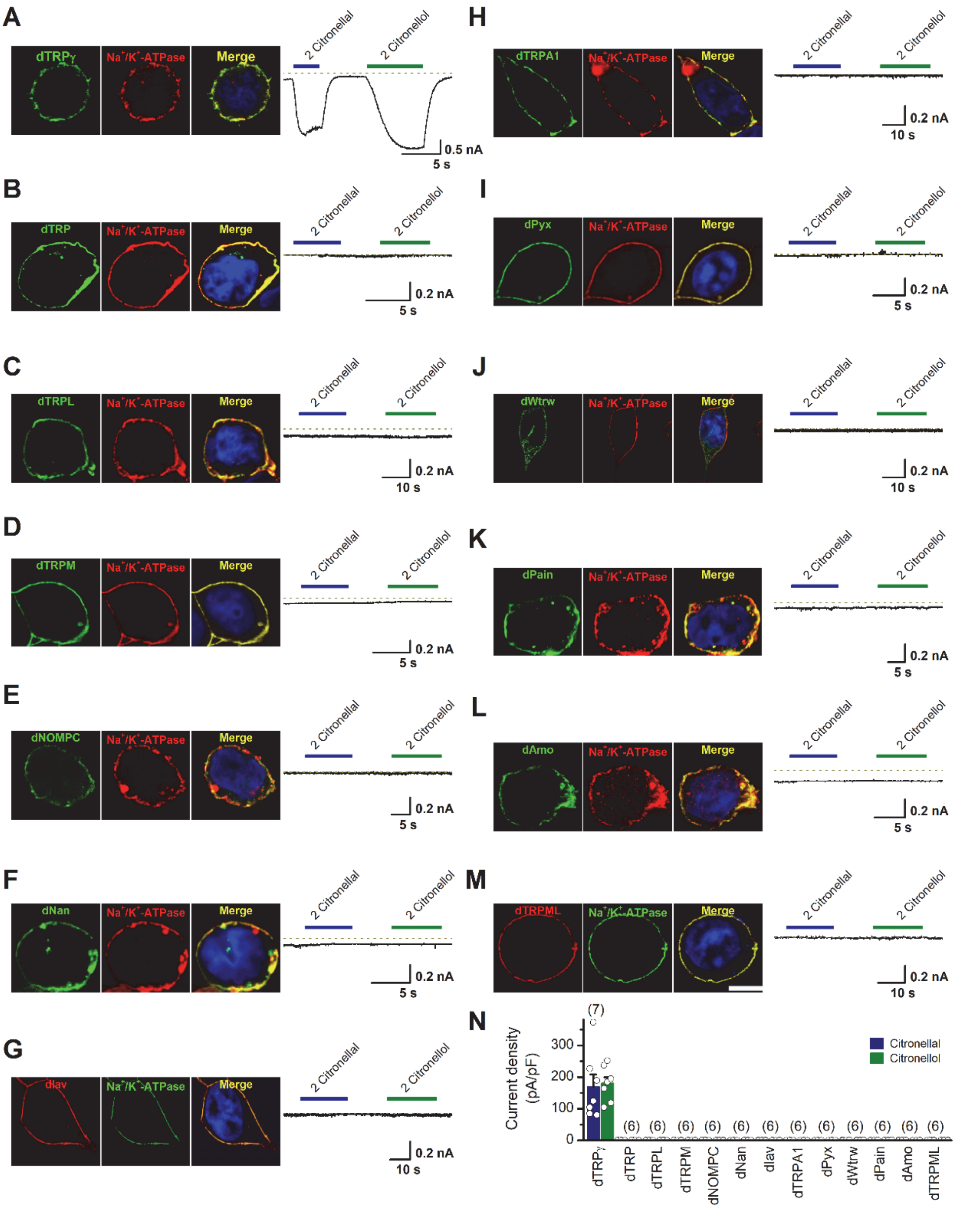

Figure S7. Of 13 TRP channels in *Drosophila*, only TRPγ responds to citronellal and citronellol.

(**A-M**) Confocal microscopic images depicting the expression and localization of *Drosophila* TRP channels (left), Na^+-K^+-ATPase (middle) and Nucleus (blue) by DAPI. HEK 293T cells were transfected with dTRP channels fused with the indicated fluorescent protein and Na^+/K^+-ATPase was stained by antibody and served as a plasma membrane marker. Whole-cell recordings were made at V_h = -60 mV from HEK 293T cells transfected with each dTRP channel. 2 mM citronellal and 2 mM citronellol were

sequentially applied to examine the responses of individual dTRP channels. *n* = 6 per channel. (Scale bar, 10 μm). The dotted line indicates zero current level. (**N**) Summary plot of current density.

Supplementary Figure 8

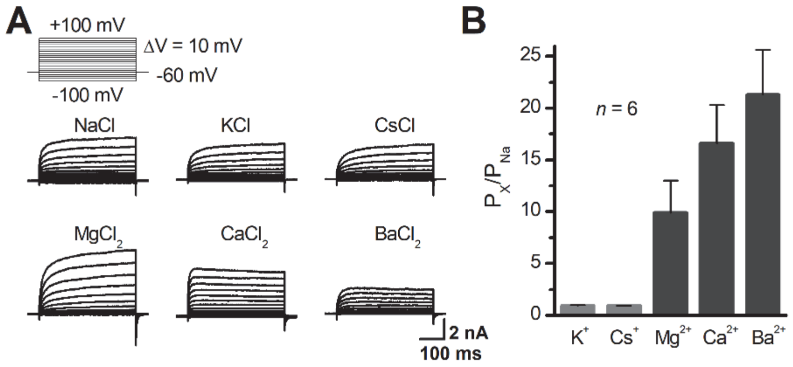

Figure S8. Ion permeation properties of *Drosophila* TRPγ.
(**A**) Representative whole-cell currents of dTRPγ–expressing HEK 293T cells evoked by a family of voltage pulses ranging from -100 mV to 100 mV with a 10-mV increment in the presence of citronellal (0.5 mM), with bath solution containing different cations as indicated. Pipette solutions contained 140 mM NaCl. (**B**) Permeability ratios (P_X/P_{Na}) calculated from reversal potential shifts in (**A**).

Supplementary Figure 9

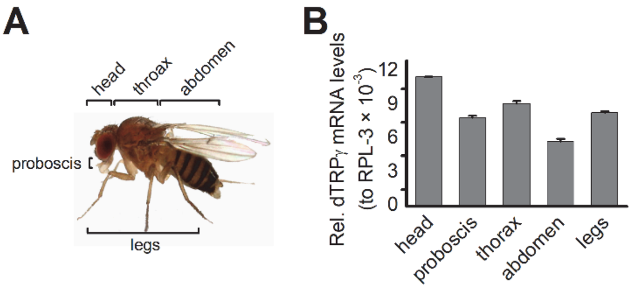

Figure S9. Distribution of *Drosophila trpγ* gene.
(**A**) Schematic picture for indicating the proboscis, head, thorax, abdomen, and legs of *D. melanogaster*. (**B**) Quantitative analysis of the relative mRNA levels (normalized by 10^{-3} fold of RPL-3) of dTRPγ in different parts of *D. melanogaster* shown in (**A**).

Supplementary Figure 10

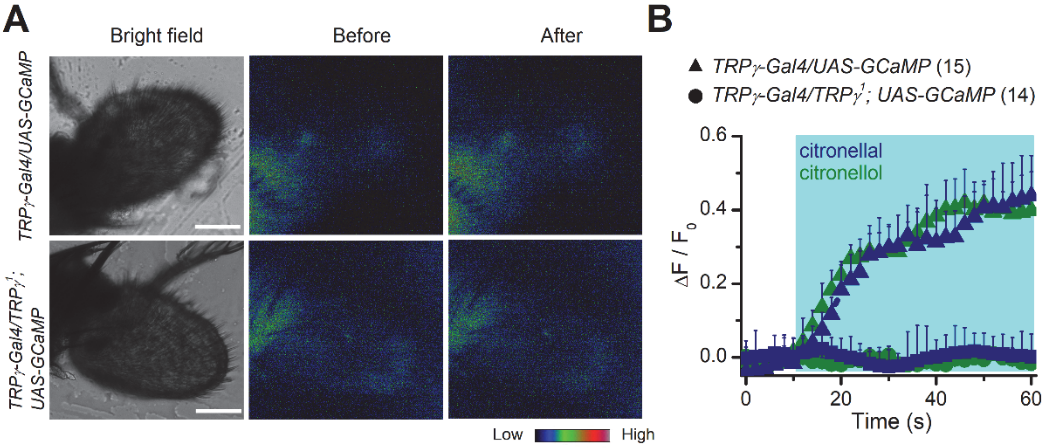

Figure S10. dTRPγ mediates the calcium elevation in response to repellents in *Drosophila* antenna.

(**A**) Responses of *Drosophila* antennas of WT (*TRPγ-Gal4/UAS-GCaMP*) and TRPγ-KO (*TRPγ-Gal4/TRPγ^{1}; UAS-GCaMP*) to citronellol (4 mM) and citronellal (4 mM) measured by GCaMP fluorescence in the presence of 2 mM extracellular Ca^{2+}. Images shows the GCaMP fluorescence intensity before and after application of citronellol. Scale bar indicates 50 μm. (**B**) Time courses of the relative changes of fluorescence were plotted. Error bars indicate SEM.

Supplementary Figure 11

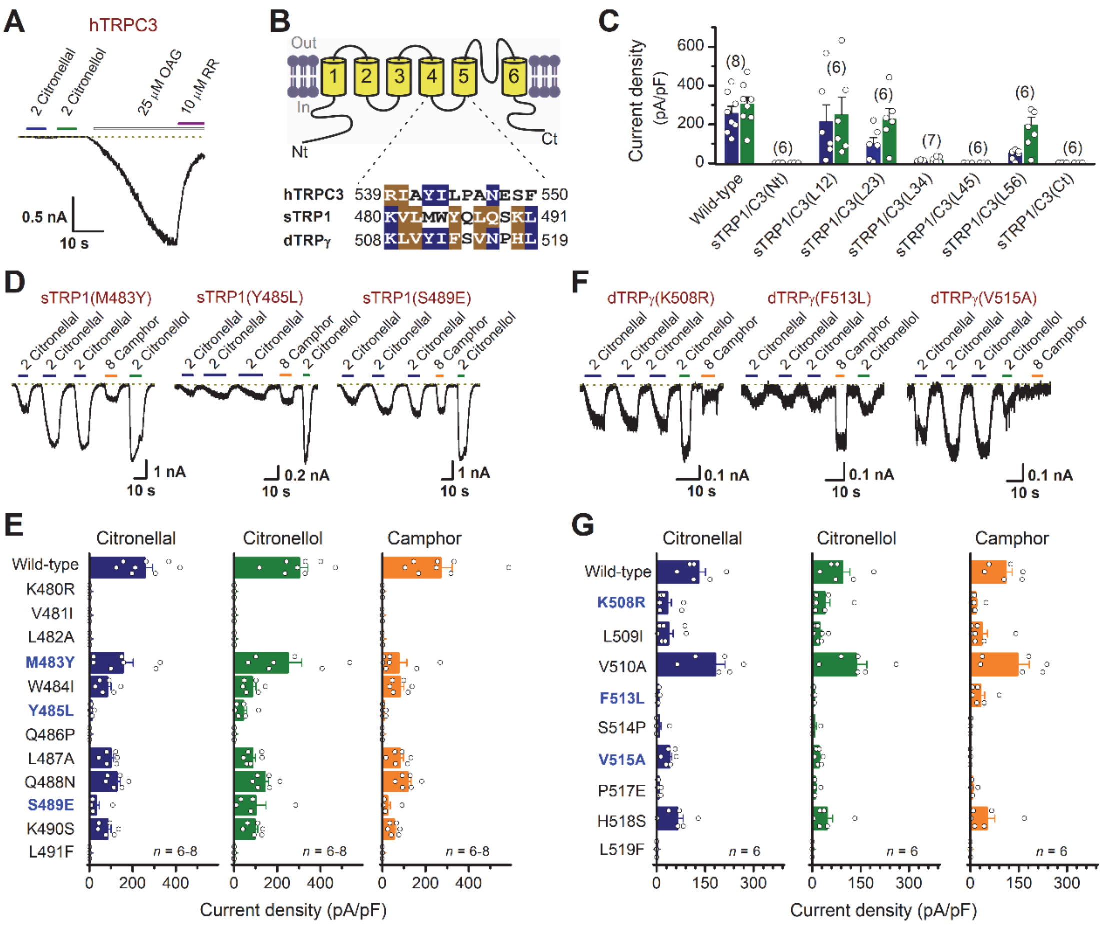

Figure S11. Residues within the S4-S5 linker mediates the activation of sTRP1/dTRPγ channel by repellents.
(**A**) Representative whole-cell recordings from hTRPC3-expressing HEK 293T cells in response to 2 mM citronellal, and 2 mM citronellol, 25 μM OAG, and the combination of 25 μM OAG and 10 μM RR. (**B**) Amino acid alignments of the linker S4-S5 among hTRPC3, sTRP1 and dTRPγ, with identical residues shaded in blue and similar in brown. (**C**) Summary data of current densities of sTRP1 mutants at -60 mV evoked by the indicated repellents obtained by whole-cell recordings. sTRP1 mutants with the N terminus (Nt), linker of transmembrane domains 1 and 2 (L12), 2 and 3 (L23), 3 and 4 (L34), 4 and 5 (L45), 5 and 6 (L56), and the C terminus (Ct) swapped by the cognate segments of human TRPC3, respectively (*n* = 6-8). (**D**) Representative whole-cell recordings showing the responses of sTRP1(M483Y), sTRP1(Y485L), and sTRP1(S489E) to the indicated repellents. (**E**) Current densities of the mutant channels compared with that of wild-type sTRP1 (*n* = 6-8). (**F**) Representative whole-cell currents recorded from HEK 293T cells that expressed dTRPγ(K508R), dTRPγ(F513L), and dTRPγ(V515A) in response to repellents, respectively. (**G**) Summary plot of current densities of dTRPγ mutations (*n* = 6).

Supplementary Figure 12

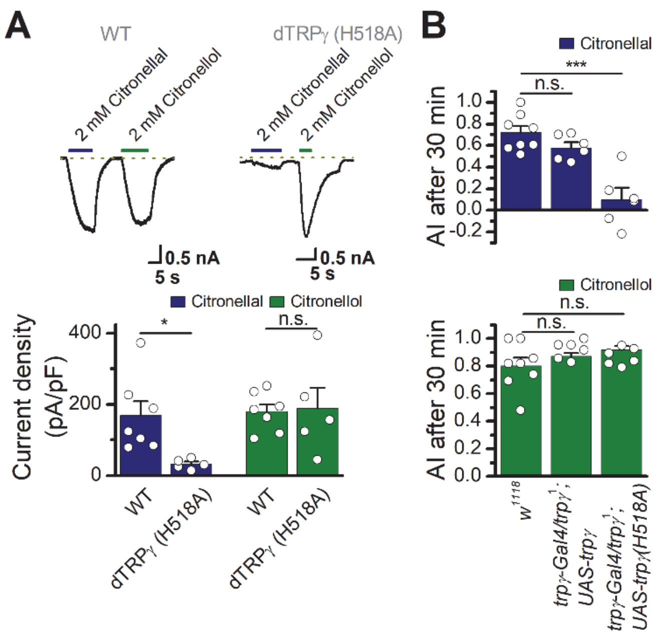

Figure S12. Rescue of the *trpγ¹* phenotype with either *dtrpγ (WT)* or *dtrpγ (H518A)* transgenes using the *GAL4/UAS* system

(**A**) Representative whole-cell recordings from HEK293T cells expressing dTRPγ (WT) or dTRPγ (H518A) in response to 2 mM citronellal and 2 mM citronellol, respectively. V_h = -60 mV. Below is the summary plot of current densities. **p* = 0.017 by unpaired Student's *t* test. (**B**) Reconstitution of the *UAS-trpγ (WT)* or *UAS-trpγ (H518A)* in *dTRPγ-knock out* (KO) strain to examine the avoidance responses. ****p* = 1.39E-05 by ANOVA.

Supplementary Table 1 *Drosophila* mutants

Mutants	RRID	Mutants	RRID
$trp\gamma^{1}$	BDSC_BS64311	$trp\gamma^{Gal4}$	BDSC_BS64313
$trpl^{302}$	BDSC_BS31433	$trpm^{2}$	BDSC_BS35527
$trpa1^{1}$	BDSC_BS36342	$nompC^{3}$	BDSC_BS42258
iav^{3621}	BDSC_BS24768	trp^{74}	BDSC_BS24895
wtrw-RNAi	BDSC_BS31292	***pyx-RNAi***	BDSC_BS31297
$trpml^{1}$	BDSC_BS28992	$pain^{EP2251}$	BDSC_BS31432
nan^{GAL4}	BDSC_BS68205		

Supplementary Table 2 Primers designed for cloning s*TRP1-6*

Gene	Forward Primer (from 5′ to 3′)	Reverse Primer (from 5′ to 3′)
strp1	AACACGTCACTGCTGTGT	TCCATTATTCTTCGGGAAATCTGAG
	GAAGATCTGCCACCATGTCTGACGAGATAAAGTGTCAAC	TCCCCCGGGTTATTCTTCGGGAAATCTGAGAT
strp2	CGGGGTACCGCCACCATGGAGAATGAAGACGAATTCCAGC	CCGCTCGAGTTATAAATAATCATCATCATCTATGTCATCATC
strp3	GAAGATCTGCCACCATGGGAGAATATCCTTTATCGTG	ACGCGTCGACTCACGTTCCTGGTTTAAAAGGTG
strp4	ACATTCTTCCATAAGGAAGTCGT	GTCAACATCATCAGTAACGTGTCC
	GCTCTTAACTTGACTGTTCTTAAGC	GTGAATGAACCATCATTCTCTCTAG
strp5	GAGATGCTGATCAATCATC	TGACACTTCCTTGAGAGTG
	GTATGCCATCAGGGAAGGTG	CCTTGAGAGTGCAGTAGTCG
strp6	CGACATGTCATAATGGTGTT	CCGATGATTCAAGATCAGTACT
	CCGGAATGAATAATGCATCTAC	GCGTACTGTATGCTAGAGTGT
	CCGTGAATATAATGGAATGAAT	CGATATTCTTGCCAAGATGGT

Supplementary Table 3. Gene specific primers used in qRT-PCR

Gene	Forward Primer (from 5′ to 3′)	Reverse Primer (from 5′ to 3′)
strp1	GCGTTACCTCGAATGGAAGAAA	TGCGGGTGGATGTGAAGATAAT
β-actin	GGTATAGTGACAAATTGGGATG	TTGCCTTAGGATTCAGTGGG
dtrpγ	GGCCACCCAGAAGCAACTAC	CCTAACTGCAAGGTTGACCGA
RPL-3	GCTAAGCTGTCGCACAAATG	GTTCGATCCGTAACCGATGT

Supplementary Table 4. TRP channels of some representative species

Channels	species	GeneBank® accession number
**TRPC1**	*Homo sapiens*	NP_001238774.1
**TRPC3**	*Homo sapiens*	NP_001124170.1
**TRPC4**	*Homo sapiens*	NP_057263.1
**TRPC5**	*Homo sapiens*	NP_036603.1
**TRPC6**	*Homo sapiens*	NP_004612.2
**TRPC7**	*Homo sapiens*	NP_065122.1
**TRPV1**	*Homo sapiens*	NP_542437.2
**TRPV2**	*Homo sapiens*	NP_057197.2
**TRPV3**	*Homo sapiens*	NP_001245134.1
**TRPV4**	*Homo sapiens*	NP_067638.3
**TRPV5**	*Homo sapiens*	NP_062815.3
**TRPV6**	*Homo sapiens*	NP_061116.5
**TRPM1**	*Homo sapiens*	NP_001238949.1
**TRPM2**	*Homo sapiens*	NP_001307279.2
**TRPM3**	*Homo sapiens*	NP_001007472.2
**TRPM4**	*Homo sapiens*	NP_060106.2
**TRPM5**	*Homo sapiens*	NP_055370.1
**TRPM6**	*Homo sapiens*	NP_060132.3
**TRPM7**	*Homo sapiens*	NP_060142.3
**TRPM8**	*Homo sapiens*	NP_076985.4
**TRPA1**	*Homo sapiens*	NP_015628.2
**TRPML1**	*Homo sapiens*	NP_065394.1
**TRPML2**	*Homo sapiens*	NP_694991.2
**TRPML3**	*Homo sapiens*	NP_060768.8
**PKD2**	*Homo sapiens*	NP_000288.1
**PKD2L1**	*Homo sapiens*	NP_057196.2
**PKD2L2**	*Homo sapiens*	NP_001287850.1
**TRPC1**	*Mus musculus*	NP_035773.2
**TRPC2**	*Mus musculus*	NP_001103367.1
**TRPC3**	*Mus musculus*	NP_062383.2
**TRPC4**	*Mus musculus*	NP_058680.1
**TRPC5**	*Mus musculus*	NP_033454.1
**TRPC6**	*Mus musculus*	NP_038866.2
**TRPC7**	*Mus musculus*	NP_036165.1
**TRPV1**	*Mus musculus*	NP_001001445.1
**TRPV2**	*Mus musculus*	NP_035836.2

**TRPV3**	*Mus musculus*	NP_659567.2
**TRPV4**	*Mus musculus*	NP_071300.2
**TRPV5**	*Mus musculus*	NP_001007573.1
**TRPV6**	*Mus musculus*	NP_071858.3
**TRPM1**	*Mus musculus*	NP_001034193.2
**TRPM2**	*Mus musculus*	NP_612174.2
**TRPM3**	*Mus musculus*	NP_001030316.1
**TRPM4**	*Mus musculus*	NP_780339.2
**TRPM5**	*Mus musculus*	NP_064673.2
**TRPM6**	*Mus musculus*	NP_700466.1
**TRPM7**	*Mus musculus*	NP_067425.2
**TRPM8**	*Mus musculus*	NP_599013.1
**TRPA1**	*Mus musculus*	NP_808449.1
**TRPML1**	*Mus musculus*	NP_444407.1
**TRPML2**	*Mus musculus*	NP_080932.2
**TRPML3**	*Mus musculus*	NP_598921.1
**PKD2**	*Mus musculus*	NP_032887.3
**PKD2L1**	*Mus musculus*	NP_852087.2
**PKD2L2**	*Mus musculus*	NP_058623.2
**TRPC1**	*Gallus gallus*	NP_001004409.1
**TRPC3**	*Gallus gallus*	NP_001244208.1
**TRPC4**	*Gallus gallus*	XP_417089.3
**TRPC5**	*Gallus gallus*	XP_420310.3
**TRPC6**	*Gallus gallus*	XP_417184.4
**TRPC7**	*Gallus gallus*	XP_040503162.1
**TRPV1**	*Gallus gallus*	NP_989903.1
**TRPV2**	*Gallus gallus*	XP_004946743.1
**TRPV3**	*Gallus gallus*	XP_040506402.1
**TRPV4**	*Gallus gallus*	NP_990023.1
**TRPV6**	*Gallus gallus*	XP_004938199.2
**TRPM1**	*Gallus gallus*	XP_040562765.1
**TRPM2**	*Gallus gallus*	XP_040561816.1
**TRPM3**	*Gallus gallus*	XP_015135815.1
**TRPM5**	*Gallus gallus*	XP_003641369.3
**TRPM6**	*Gallus gallus*	XP_025000684.2
**TRPM7**	*Gallus gallus*	XP_040562330.1
**TRPM8**	*Gallus gallus*	NP_001007083.1
**TRPA1**	*Gallus gallus*	NP_001305389.1

**MCOLN1**	*Gallus gallus*	XP_015128608.2
**MCOLN2**	*Gallus gallus*	XP_422368.2
**MCOLN3**	*Gallus gallus*	XP_040560794.1
**PKD2**	*Gallus gallus*	NP_001026311.1
**PKD2L1**	*Gallus gallus*	XP_040559089.1
**PKD2L2**	*Gallus gallus*	XP_414617.4
**TRPC1.L**	*Xenopus laevis*	NP_001083819.1
**TRPC1.S**	*Xenopus laevis*	XP_018121270.1
**TRPC3.S**	*Xenopus laevis*	XP_041435519.1
**TRPC6.L**	*Xenopus laevis*	XP_041438627.1
**TRPV1.S**	*Xenopus laevis*	NP_001177322.1
**TRPV2.S**	*Xenopus laevis*	XP_018104301.1
**TRPV3.L**	*Xenopus laevis*	XP_018101159.2
**TRPV4.L**	*Xenopus laevis*	XP_018116507.1
**TRPV5.L**	*Xenopus laevis*	XP_018080408.1
**TRPV6.L**	*Xenopus laevis*	XP_018080412.1
**TRPM2.L**	*Xenopus laevis*	XP_041432692.1
**TRPM3.L**	*Xenopus laevis*	XP_041420158.1
**TRPM4.L**	*Xenopus laevis*	XP_041425588.1
**TRPM6.L**	*Xenopus laevis*	XP_018114314.1
**TRPM7.L**	*Xenopus laevis*	NP_001177950.3
**TRPM8.L**	*Xenopus laevis*	NP_001155066.1
**TRPA1.L**	*Xenopus laevis*	XP_018097008.1
**MCOLN1.L**	*Xenopus laevis*	NP_001085687.1
**MCOLN2.L**	*Xenopus laevis*	NP_001085127.1
**MCOLN3.S**	*Xenopus laevis*	NP_001085683.1
**PKD2.L**	*Xenopus laevis*	XP_018107809.1
**TRPC1**	*Danio rerio*	NP_001185590.1
**TRPC2b**	*Danio rerio*	NP_001025337.1
**TRPC3**	*Danio rerio*	NP_001276813.2
**TRPC4a**	*Danio rerio*	NP_001276811.1
**TRPC4b**	*Danio rerio*	NP_001276810.1
**TRPC5a**	*Danio rerio*	NP_001314676.1
**TRPC6a**	*Danio rerio*	NP_001025453.1
**TRPC7a**	*Danio rerio*	NP_001276808.1
**TRPC7b**	*Danio rerio*	NP_001276815.1
**TRPV1**	*Danio rerio*	NP_001119871.1
**TRPV4**	*Danio rerio*	NP_001036195.1

**TRPV6**	*Danio rerio*	NP_001001849.1
**TRPM1a**	*Danio rerio*	NP_001303302.1
**TRPM2**	*Danio rerio*	NP_001275746.1
**TRPM4a**	*Danio rerio*	NP_001275744.2
**TRPM4b.2**	*Danio rerio*	NP_001269082.1
**TRPM5**	*Danio rerio*	NP_001121711.1
**TRPM6**	*Danio rerio*	NP_001238760.1
**TRPM7**	*Danio rerio*	NP_001025232.1
**TRPA1a**	*Danio rerio*	NP_001007066.1
**TRPA1b**	*Danio rerio*	NP_001007067.1
**TRPN1**	*Danio rerio*	NP_899192.1
**MCOLN1a**	*Danio rerio*	NP_001315094.1
**MCOLN2**	*Danio rerio*	NP_957442.1
**PKD2**	*Danio rerio*	NP_001002310.1
**TRP**	*Drosophila melanogaster*	NP_476768.1
**TRPL**	*Drosophila melanogaster*	NP_476895.1
**TRPγ**	*Drosophila melanogaster*	NP_609802.1
**NAN**	*Drosophila melanogaster*	NP_648696.2
**IAV**	*Drosophila melanogaster*	NP_572353.1
**TRPM**	*Drosophila melanogaster*	NP_001137672.2
**TRPA1**	*Drosophila melanogaster*	NP_001261602.1
**PAIN**	*Drosophila melanogaster*	NP_611979.1
**WTRW**	*Drosophila melanogaster*	NP_731193.1
**PYX**	*Drosophila melanogaster*	NP_612015.1
**NOMPC**	*Drosophila melanogaster*	NP_001097089.2
**TRPML**	*Drosophila melanogaster*	NP_649145.1
**PKD2**	*Drosophila melanogaster*	NP_609561.2
**TRPC3.L**	*Parasteatoda tepidariorum*	XP_020999857.1
**TRPC4.L**	*Parasteatoda tepidariorum*	XP_015927173.1
**TRPC7.L**	*Parasteatoda tepidariorum*	XP_020999858.1
**TRPV5**	*Parasteatoda tepidariorum*	XP_021004170.1
**TRPV5.L**	*Parasteatoda tepidariorum*	XP_015907938.1
**TRPM1**	*Parasteatoda tepidariorum*	XP_021001386.1
**TRPM2**	*Parasteatoda tepidariorum*	XP_015912591.1
**TRPM2.L**	*Parasteatoda tepidariorum*	XP_015907226.1
**TRPM3**	*Parasteatoda tepidariorum*	XP_015924394.1
**TRPA1**	*Parasteatoda tepidariorum*	XP_015910182.1
**TRPγ.L**	*Parasteatoda tepidariorum*	XP_015916803.1

**TRPL**	*Parasteatoda tepidariorum*	XP_015916852.1
**TRP-1**	*Caenorhabditis elegans*	NP_001367402.1
**TRP-2**	*Caenorhabditis elegans*	NP_497311.2
**OSM-9**	*Caenorhabditis elegans*	NP_500372.1
**OCR-1**	*Caenorhabditis elegans*	NP_001335506.1
**OCR-2**	*Caenorhabditis elegans*	NP_501380.1
**OCR-3**	*Caenorhabditis elegans*	NP_510520.4
**OCR-4**	*Caenorhabditis elegans*	NP_501172.2
**GTL-1**	*Caenorhabditis elegans*	NP_502111.3
**GTL-2**	*Caenorhabditis elegans*	NP_502118.4
**GON-2**	*Caenorhabditis elegans*	NP_492315.4
**CED-11**	*Caenorhabditis elegans*	NP_499021.1
**TRPA1**	*Caenorhabditis elegans*	NP_502249.3
**TRPA2**	*Caenorhabditis elegans*	NP_492031.1
**TRP-4**	*Caenorhabditis elegans*	NP_493429.2
**CUP-5**	*Caenorhabditis elegans*	NP_001022721.2
**PKD-2**	*Caenorhabditis elegans*	NP_502838.3

Supplementary Movie S1. Movie showing the avoidance response assay in scorpions.

To test the scorpion avoidance response, two pieces of gauze (8 cm × 8 cm) were pre-immersed with 2 ml water (control) or repellents (citronellol 50% vol/vol; citronellal 50% vol/vol; DEET 100%; menthol 500 mM; and camphor 500 mM) and placed at one side of the container (100 cm × 50 cm × 30 cm) with a layer of clean sand. Each gauze was covered by a piece of clay tile. For each trial, 100 scorpions were placed at the center of the container covered with a piece of glass (100 cm × 50 cm). The numbers of scorpions under and outside the tiles were separately counted after about 1 h. After each trial, sand and tiles were renewed and container was cleaned thoroughly.
